# British Obesity and Metabolic Surgery Society Guidelines on perioperative and postoperative biochemical monitoring and micronutrient replacement for patients undergoing bariatric surgery—2020 update

**DOI:** 10.1111/obr.13087

**Published:** 2020-08-02

**Authors:** Mary O'Kane, Helen M. Parretti, Jonathan Pinkney, Richard Welbourn, Carly A. Hughes, Jessica Mok, Nerissa Walker, Denise Thomas, Jennifer Devin, Karen D. Coulman, Gail Pinnock, Rachel L. Batterham, Kamal K. Mahawar, Manisha Sharma, Alex I. Blakemore, Iris McMillan, Julian H. Barth

**Affiliations:** ^1^ Dietetic Department Leeds Teaching Hospitals NHS Trust Leeds UK; ^2^ Norwich Medical School University of East Anglia Norwich UK; ^3^ Faculty of Health and Human Sciences Peninsula Schools of Medicine and Dentistry Plymouth UK; ^4^ Department of Endocrinology Plymouth Hospitals NHS Trust Plymouth UK; ^5^ Department of Upper GI and Bariatric Surgery, Musgrove Park Hospital Taunton and Somerset NHS Foundation Trust Taunton UK; ^6^ Fakenham Weight Management Service Fakenham Medical Practice Fakenham UK; ^7^ Centre for Obesity Research, Rayne Institute, Department of Medicine University College London London UK; ^8^ School of Biosciences Sutton Bonington Campus, University of Nottingham Nottingham UK; ^9^ Department of Nutrition and Dietetics Portsmouth Hospitals NHS Trust Portsmouth UK; ^10^ Specialist Weight Management Service Betsi Cadwaladr University Health Board Wales UK; ^11^ Population Health Sciences Bristol Medical School. University of Bristol Bristol UK; ^12^ Obesity and Bariatric Surgery Service Southmead Hospital, North Bristol NHS Trust Bristol UK; ^13^ Freelance Dietitian Somerset UK; ^14^ Bariatric Centre for Weight Management and Metabolic Surgery, UCLH University College London Hospital (UCLH) London UK; ^15^ National Institute of Health Research UCLH Biomedical Research Centre London UK; ^16^ Department of General Surgery Sunderland Royal Hospital Sunderland UK; ^17^ Department of Clinical Biochemistry & Bariatric Surgery Homerton University Hospital NHS Trust London UK; ^18^ Department of Life Sciences Brunel University London UK; ^19^ Department of Medicine Imperial College London UK; ^20^ Patient Representative Edinburgh UK; ^21^ Department of Chemical Pathology & Metabolic Medicine Leeds Teaching Hospitals NHS Trust Leeds UK

**Keywords:** bariatric surgery, guidelines, nutrition, micronutrients

## Abstract

Bariatric surgery is recognized as the most clinically and cost‐effective treatment for people with severe and complex obesity. Many people presenting for surgery have pre‐existing low vitamin and mineral concentrations. The incidence of these may increase after bariatric surgery as all procedures potentially cause clinically significant micronutrient deficiencies. Therefore, preparation for surgery and long‐term nutritional monitoring and follow‐up are essential components of bariatric surgical care. These guidelines update the 2014 British Obesity and Metabolic Surgery Society nutritional guidelines. Since the 2014 guidelines, the working group has been expanded to include healthcare professionals working in specialist and non‐specialist care as well as patient representatives. In addition, in these updated guidelines, the current evidence has been systematically reviewed for adults and adolescents undergoing the following procedures: adjustable gastric band, sleeve gastrectomy, Roux‐en‐Y gastric bypass and biliopancreatic diversion/duodenal switch. Using methods based on Scottish Intercollegiate Guidelines Network methodology, the levels of evidence and recommendations have been graded. These guidelines are comprehensive, encompassing preoperative and postoperative biochemical monitoring, vitamin and mineral supplementation and correction of nutrition deficiencies before, and following bariatric surgery, and make recommendations for safe clinical practice in the U.K. setting.

**Abbreviations**
25OHD: 25‐hydroxyvitamin D
AGB: adjustable gastric band
BOMSS: British Obesity and Metabolic Surgery Society
BPD/DS: duodenal switch
CT: non‐randomized controlled trials
EL: evidence level
FPG: fasting plasma glucose
GPP: good practice point
OAGB/MGB: one anastomosis gastric bypass/mini gastric bypass
PIVKA‐II: protein induced by vitamin K absence or antagonism
PTH: parathyroid hormone
RCT: randomized controlled trial
ROS: Royal Osteoporosis Society
RYGB: Roux‐en‐Y gastric bypass
SADIs: single anastomosis duodenal ileal bypass with sleeve gastrectomy
SG: sleeve gastrectomy
SIGN: Scottish Intercollegiate Guidelines Network

## 1 INTRODUCTION

Bariatric surgery is recognized as the most clinically and cost‐effective treatment option for people with severe and complex obesity.^1^ All bariatric surgery procedures impact on nutrition to varying degrees and can potentially cause clinically significant deficiencies of micronutrients.^2^, ^3^, ^4^

The British Obesity and Metabolic Surgery Society (BOMSS) published its first nutritional guidelines in 2014 following a review of U.K. clinicians, which showed widespread variation in nutritional monitoring and vitamin and mineral supplementation both pre‐ and post‐surgery.^5^, ^6^ This is an update and review of those guidelines. Since the 2014 guidelines, a number of other nutritional guidelines for the care of people who undergo bariatric surgery have been published.^3^, ^4^, ^7^, ^8^, ^9^, ^10^, ^11^ However, not all have been specifically related to micronutrition, had dietetic involvement and included either primary care or patient representatives. In addition, previous guidance aimed at the management of adolescents undergoing bariatric surgery has only included general nutritional recommendations,^12^, ^13^, ^14^ despite this vulnerable group being at high risk of developing nutritional deficiencies.^15^, ^16^, ^17^, ^18^, ^19^ Until recent guidelines by Shawe et al,^11^ there has been little detail included in previous guidelines on recommendations for the nutritional management of pregnancy post‐surgery. In summary, there are no up‐to‐date, comprehensive U.K. guidelines for the perioperative and postoperative nutritional care of patients undergoing bariatric surgery.

In this update of BOMSS nutritional guidelines,^5^ we have aimed to systematically review the current evidence for preoperative and postoperative biochemical monitoring and micronutrient replacement for adolescents and adults undergoing bariatric surgery, including pregnancy, and to update the recommendations for safe practice in the U.K. setting. We have focussed on the procedures: adjustable gastric band (AGB), sleeve gastrectomy (SG), Roux‐en‐Y gastric bypass (RYGB) and duodenal switch (BPD/DS), as these were the most common procedures being undertaken at the time of the literature search. The aim of this work was to produce a set of guidelines that are comprehensive but remain accessible to healthcare professionals involved in preparation and aftercare of people undergoing bariatric surgery, in particular those working in the United Kingdom.

## 2 METHODS

Members of the original BOMSS nutritional guidelines were involved in the update with additional members selected on the basis of their expertise and experience in supporting people who undergo bariatric surgery. In addition, two patient representatives were included. The membership therefore included six dietitians (MO, GP, NW, DT, KC and JD), two general practitioners (HP and CH), three physicians (JP, RB and JM), two chemical pathologists (JB and MS), two surgeons (RWand KM) and two patient representatives (AB and IM). These guidelines aimed to address four main areas of clinical management for patients undergoing bariatric surgery.

What are the clinical recommendations that are appropriate for:

- Preoperative nutritional screening/or monitoring before bariatric surgery;
- Postoperative nutritional screening and/or monitoring after bariatric surgery;
- Vitamin and mineral supplementation to prevent nutritional deficiencies following bariatric surgery;
- Treatment of nutritional deficiencies before/after bariatric surgery?

In order to update the evidence base from the 2014 BOMSS guidelines,^5^ the Cochrane library, NHS Evidence and the HDAS database platform, specifically Medline, PubMed, Cinahl and Embase databases, were searched from 2014 to January 2018 to identify studies and publications related to the nutritional care of adult and/or adolescent patients pre‐ or post‐bariatric surgery. This included studies on the prevention, incidence, prevalence or treatment of nutritional deficiencies pre‐ and/or post‐bariatric surgery. For pragmatic reasons, the searches were restricted to publications written in English. A sample search strategy can be found in Table S1. We also screened any publications suggested by members of the guidelines group based on their knowledge of their own areas of expertise.

Studies were included if they addressed the care of adults or adolescents who were planning or had undergone a bariatric surgical procedure. We focused on AGB, SG, RYGB and BPD/DS as these were the most common procedures undertaken at the time of the literature search. To be included, papers were required to report data on the nutritional care of people undergoing bariatric surgery. This included both pre‐ and post‐bariatric surgery care and both nutritional monitoring and treatment to manage nutritional deficiencies. We excluded studies in one anastomosis gastric bypass/mini gastric bypass (OAGB/MGB) and single anastomosis duodenal ileal bypass with sleeve gastrectomy (SADIS), because of the scarcity of long‐term data at that time and excluded studies on groups with conditions that in themselves may lead to nutritional problems, such as coeliac disease. We included any study type as well as previous relevant clinical guidelines but excluded letters, editorials and conference abstracts.

Titles and abstracts were screened for inclusion (MO, HP, CH, JP, RB, JB, JM, EA, RW, KM, MS and GP). Full papers were screened by two independent reviewers (MO and HP). All of the author group, except the patient representatives, were then divided into pairs of independent reviewers, and a standardized data extraction form was used to characterize the population, intervention, control groups (where appropriate) and study outcomes and to appraise the quality of the study. Disagreements were resolved by discussion or by a third reviewer. Study quality was assessed using an established quality assessment tool appropriate for each study type.^20^, ^21^, ^22^, ^23^, ^24^, ^25^, ^26^, ^27^ (See Table S2 for details of the quality assessment tools).

Data extracted from the studies were initially synthesized by MO with discussion with members of the guidelines group on specific areas of expertise where needed. Early proposed recommendations were circulated amongst the guideline group for iterative discussion and review. Following this, the contributing evidence and recommendations were graded based on Scottish Intercollegiate Guidelines Network^28^ (SIGN) methodology (see Table 1) by two independent reviewers (MO and HP). Draft recommendations together with their grading were then circulated within the guideline group in an iterative process until consensus was achieved on both the recommendations and grading. Although we included previously published guidelines in our literature review, we graded those recommendations as being expert opinion unless we had reviewed the original articles cited by the authors. Just prior to submission, “Clinical practice guidelines for the perioperative nutrition, metabolic, and nonsurgical support of patients undergoing bariatric procedures ‐ 2019 update were published so were included.”^4^

**TABLE 1:** Levels of evidence, grades of recommendations and good practice points (SIGN 50^28^)

**Levels of evidence**	
1++	High‐quality meta‐analyses, systematic reviews of RCTs or RCTs with a very low risk of bias
1+	Well‐conducted meta‐analyses, systematic reviews or RCTs with a low risk of bias
1‐	Meta‐analyses, systematic reviews or RCTs with a high risk of bias
2++	High‐quality systematic reviews of case control or cohort studies High‐quality case‐control or cohort studies with a very low risk of confounding or bias and a high probability that the relationship is causal
2+	Well‐conducted case‐control or cohort studies with a low risk of confounding or bias and a moderate probability that the relationship is causal
2‐	Case‐control or cohort studies with a high risk of confounding or bias and a significant risk that the relationship is not causal
3	Non‐analytical studies, e.g., case reports and case series
4	Expert opinion
**Grades of recommendation** The grade of recommendation relates to the strength of supporting evidence and not the clinical importance of the recommendation	
A	At least one meta‐analysis, systematic review or RCT rated as 1++ and directly applicable to the target population; or A body of evidence consisting principally of studies rated as 1+, directly applicable to the target population and demonstrating overall consistency of results
B	A body of evidence including studies rated as 2++, directly applicable to the target population and demonstrating overall consistency of results; or Extrapolated evidence from studies rated as 1++ or 1+
C	A body of evidence including studies rated as 2+, directly applicable to the target population and demonstrating overall consistency of results; or
D	Extrapolated evidence from studies rated as 2++ Evidence level 3 or 4; or Extrapolated evidence from studies rated as 2+
**Good practice point**	
GPP	Recommended best practice based on the clinical experience of the guideline development group

Abbreviation: RCTs, randomized controlled trials.

## 3 RESULTS

The searches identified 601 studies, 510 of which were identified through database searches and 91 from other sources. One hundred and seventy‐one full papers were screened, and 139 were included. The main reasons papers were excluded were that no relevant nutritional data were reported or that the study population did not include people who had undergone bariatric surgery. The 32 full papers removed with reasons for exclusion are listed as Supporting Information in Table S3.

## 4 SUMMARY OF THE STUDIES

Pre‐bariatric surgery studies included seven cross‐sectional and two cohort studies. Pre‐ and post‐bariatric surgery studies included one non‐randomized controlled trials (CTs), one randomized controlled trials (RCTs), 20 cohort studies, one case control study, seven systematic reviews, eight narrative reviews and seven clinical practice guidelines. Post‐surgery only studies included two CTs, six RCTs, 30 cohort studies, two case control studies, seven cross‐sectional studies, one case series, 10 case studies, one survey, nine systematic reviews, seven narrative reviews and 10 clinical practice guidelines. Eight studies/reviews included adolescents only, and one included both adolescents and adults. The rest of the papers included adults only. There were 45 studies or systematic reviews, which included SG; 74 included RYGB; 29 included BPD/DS; and 17 included AGB. Most clinical practice guidelines and narrative reviews focused on a range of procedures including AGB, SG, RYGB and BPD/DS. Studies were conducted in a variety of geographical areas including Australia, Canada, Middle East, South America and Asian countries (23 studies in total), but the majority were conducted in Europe (73 studies) or in the United States (40 studies). Follow‐up in the postoperative studies varied between 6 months and 15 years. The majority reported at 1 year with smaller numbers reported at 5, 10 or 15 years. Forty‐nine studies reported on a range of micronutrients; 18 focused on vitamin D, calcium or bone health; 17 focused on anaemia, iron, folate or vitamin B12; eight focused on nutrition issues related to neurological disorders; and seven focused on trace minerals.

## 5 OVERVIEW AND RATIONALE FOR RECOMMENDATIONS

There is a high prevalence of nutritional deficiencies in both adults and adolescents with severe and complex obesity. The most common deficiencies include anaemia, ferritin, folate, vitamin B12 and vitamin D.^15^, ^29^, ^30^, ^31^, ^32^, ^33^, ^34^, ^35^, ^36^, ^37^, ^38^, ^39^, ^40^, ^41^, ^42^, ^43^, ^44^, ^45^, ^46^, ^47^, ^48^, ^49^, ^50^ Less frequently, lower levels of thiamine,^36^, ^43^ vitamin A^50^ and zinc and copper^33^ have been reported. Following surgery, the risk of nutritional deficiencies increases because of the impact of bariatric surgery on both oral intake and absorption.

## 6 PREOPERATIVE CARE

All people should have a comprehensive nutritional assessment prior to bariatric surgery. Detailed preoperative care recommendations are listed in Table 2. A specialist dietitian, skilled in bariatric nutrition, should undertake a detailed dietary and nutritional assessment.^1^, ^2^, ^3^, ^4^, ^5^, ^13^, ^51^ Essential preoperative blood tests include screening for nutritional deficiencies, diabetes, dyslipidaemia and renal function.^2^, ^3^, ^4^, ^9^, ^10^, ^12^, ^13^, ^14^, ^52^, ^53^, ^54^ Additional discretionary tests should be considered if clinically indicated: for instance, vitamin A, zinc, copper and selenium serum levels before malabsorptive procedures such as BPD/DS,^3^, ^4^ because of the higher levels of postoperative deficiencies. (See Sections 7.4 and 7.5).

**TABLE 2:** Preoperative nutritional assessment

Preoperative nutritional assessment	Grade, evidence level, (range of evidence)
Recommendations
•All people should have a comprehensive nutritional assessment prior to bariatric surgery	Grade D EL 4
Haematinics	
•Check full blood count including haemoglobin, ferritin, folate and vitamin B12 levels	Grade B EL 2 (1+ to 4)
Vitamin D, calcium and parathyroid hormone	
•Check serum 25‐hydroxyvitamin D levels	Grade B EL 2 (2++ to 3)
•Check serum calcium levels	Grade D EL 4
•Check serum/plasma parathyroid hormone levels	Grade B EL 2 (2++ to 3)
•Seek advice from a specialist with expertise in primary hyperparathyroidism if primary hyperparathyroidism is suspected	GPP
Vitamin A, zinc, copper, selenium and malabsorptive procedures	
•Consider checking serum vitamin A levels in individuals going forward for malabsorptive procedures such as BPD/DS or where vitamin A deficiency may be suspected	GPP
•Consider checking serum zinc, copper and selenium levels in individuals going forward for malabsorptive procedures such as BPD/DS or if a deficiency is suspected	GPP
Thiamine	
•There is insufficient evidence to support a recommendation to screen an individual's thiamine levels pre surgery; however, some individuals may have low levels	Grade D EL 3 (2− to 3)
Magnesium	
•There is insufficient evidence to support a recommendation to screen an individual's magnesium level pre‐surgery	GPP
HbA_1c_, lipids, liver and renal function	
•Routinely screen HbA_1c_, lipid profile, liver and kidney function tests and treat as necessary	GPP
Correction of nutritional deficiencies preoperatively	
•Treat and correct nutritional deficiencies preoperatively as individuals have an increased risk of deficiencies postoperatively	GPP

Abbreviations: BPD/DS, duodenal switch; EL, evidence level and depicts where the majority of evidence lies; GPP, good practice point. The range of evidence level is given in brackets.

Nutritional deficiencies should be investigated and corrected as clinically indicated before surgery.^3^ The dietitian should continue to give support and education as part of preoperative preparation.^3^, ^14^, ^51^, ^54^

Many centres recommend that people follow a low calorie/low carbohydrate diet immediately prior to surgery to reduce the size of the liver.^55^ As these diets are not always nutritionally complete, a multivitamin and mineral supplement are needed.^56^

### 6.1 Haematinics

Prevalences of iron deficiency and low haemoglobin levels ranging from 0% to 47% have been reported in people going forward for bariatric surgery.^15^, ^30^, ^31^, ^32^, ^33^, ^35^, ^36^, ^37^, ^41^, ^43^, ^46^, ^49^, ^57^, ^58^, ^59^ Reports of folate deficiency before bariatric surgery have ranged between 0% and 63%,^15^, ^30^, ^34^, ^36^, ^50^, ^60^ and between 0% to 23% for vitamin B12 deficiency.^29,30,32‐34,36,40,41.43,47,50,58^

### 6.2 Vitamin D, calcium and parathyroid hormone

Vitamin D deficiency is common in people with severe and complex obesity^19^, ^29^, ^30^, ^32^, ^34^, ^38^, ^40^, ^42^, ^43^, ^44^, ^45^, ^46^, ^47^, ^48^, ^50^, ^58^, ^61^, ^62^, ^63^, ^64^, ^65^ with reports as high as 99% of participants in one study conducted by Ben‐Porat et al.^40^ A raised albumin‐adjusted serum calcium in the presence of raised parathyroid hormone (PTH) may indicate primary hyperparathyroidism, and so both PTH and calcium should be measured preoperatively.^66^ If primary hyperparathyroidism is suspected, advice should be sought from a specialist in this area.^66^

### 6.3 Vitamin A, zinc, copper, selenium and malabsorptive procedures

The reported prevalence of vitamin A deficiency where tested is low.^29^, ^38^, ^39^, ^43^, ^50^ Zinc, copper and selenium deficiencies are not commonly reported prior to surgery,^30^, ^36^, ^37^, ^67^ although De Luis et al.^33^ reported prevalences of 73.9% and 63.8% deficiency, respectively, for zinc and copper. We did not consider that there was strong evidence for routine checking of vitamin A, zinc, copper and selenium levels prior to bariatric surgery unless there is suspicion of deficiency.

Malabsorptive procedures such as BPD/DS are associated with an increased incidence of vitamin A, zinc, copper and selenium deficiencies,^68^, ^69^, ^70^, ^71^, ^72^, ^73^, ^74^ and so consideration may be given to checking these preoperatively before these procedures.

### 6.4 Thiamine

Few studies measure thiamine levels preoperatively. However, low levels have been reported in some people.^36^, ^43^ We did not consider there was strong evidence for routine checking of thiamine levels.

### 6.5 Magnesium

Few studies have measured magnesium prior to surgery.^36^, ^58^ Low prevalence of magnesium deficiency is reported.^36^, ^58^

### 6.6 HbA_1c_, lipids, liver and renal function

These guidelines do not specifically address the preoperative medical assessment of people with type 2 diabetes. However, biochemical monitoring is an important part of preoperative and postoperative care, because bariatric surgery is used to manage type 2 diabetes and its associated comorbidities.^1^, ^10^, ^75^, ^76^ Therefore, a brief summary of these important aspects of care is included here.

#### 6.6.1 Preoperative and perioperative management of diabetes

People with known type 2 diabetes should have an up‐to‐date preoperative evaluation of current glycaemic control including measurement of HbA_1c_, and glycaemic control should be optimized before surgery.^10^ Insulin and other diabetes medications should be reviewed and adjusted as appropriate.^10^ The European Association for the Study of Obesity taskforce has made detailed suggestions for perioperative management of diabetes.^10^ The preoperative HbA_1c_ is also a baseline from which the impact of bariatric surgery and subsequent medication adjustments are assessed. This assessment is essential in planning postoperative diabetes management, especially if withdrawal of insulin is planned.^10^

Preoperative consideration of diabetes aetiology is important if this is not already known, because the expected impact of bariatric surgery on glycaemic control differs between type 1 and type 2 diabetes. People with type 1 diabetes have absolute insulin deficiency, and while people with obesity and type 1 diabetes may achieve improved glycaemic control following bariatric surgery,^77^ they will not be expected to achieve glycaemic remission and insulin withdrawal. Furthermore, in one single‐centre observational study, the postoperative occurrence of both diabetic ketoacidosis and hypoglycaemia was observed in people with type 1 diabetes undergoing bariatric surgery.^78^ Therefore, people with type 1 diabetes require careful blood glucose monitoring to detect reducing insulin requirements.

The presence and extent of diabetes complications should also be noted preoperatively because long‐term monitoring of existing microvascular complications may still be required. There is also some evidence in one small case series that the abrupt glycaemic improvement following bariatric surgery can exacerbate proliferative diabetic retinopathy.^79^

Finally, people without known diabetes should routinely undergo preoperative screening for diabetes. Appropriate tests include HbA_1c_ and fasting plasma glucose (FPG) and/or a discretionary oral glucose tolerance test. Diabetes should be diagnosed according to established criteria.^80^

#### 6.6.2 Dyslipidaemia, liver and renal function

People with pre‐existing treated dyslipidaemia should undergo a preoperative fasting lipid profile, as a baseline from which to assess the subsequent effect of bariatric surgery on dyslipidaemia during follow‐up.^4^, ^81^

The most common liver abnormality in this patient group is non‐alcoholi fatty liver disease (NAFLD) and bariatric surgery results in pronounced improvements.^82^ Therefore, it is appropriate routinely to check liver function preoperatively to assess for the presence of NAFLD and as a baseline for any long‐term monitoring.

### 6.7 Correction of nutritional deficiencies preoperatively

As bariatric surgery impacts on oral intake and absorption, we recommend treatment and correction of deficiencies preoperatively.^3^ See Section 9.

## 7 POSTOPERATIVE CARE AND BIOCHEMICAL MONITORING

People should have access to lifelong monitoring following bariatric surgery to ensure that nutritional requirements are met, and risks of developing post‐bariatric surgery–related nutritional deficiencies are reduced.^1^, ^2^, ^3^, ^4^, ^8^, ^10^, ^12^, ^13^, ^14^, ^83^ The type and frequency of nutritional monitoring should reflect the bariatric procedure and may need to be individualized. The follow‐up care should remain with the bariatric surgery centre for the first 2 years.^1^, ^8^, ^83^ Following discharge, people should be offered lifelong monitoring of nutritional status at least annually as part of shared‐care management.^1^, ^8^, ^83^ Detailed recommendations are listed in Table 3.

**TABLE 3:** Postoperative care and biochemical monitoring

Postoperative care and biochemical monitoring	Grade, evidence level, range of evidence
Recommendations	
•Specialist postoperative dietetic support should be provided including individualized nutritional supplementation, support and guidance to achieve long‐term weight loss and weight maintenance	Grade D EL 4
•People who have bariatric surgery should have a postoperative follow‐up care package within the bariatric surgery service for a minimum of 2 years. This should include monitoring nutritional intake, dietary and nutritional assessment, advice and support	Grade D EL 4
•People discharged from bariatric surgery service follow‐up should undergo monitoring of nutritional status at least once a year as part of a shared care model of management	Grade D EL 4
Urea and electrolytes, renal and liver function tests	
•Monitor renal and liver function 3, 6 and 12 months in the first year and then at least annually	GPP
Haematinics	
Full blood count and ferritin	
•Check full blood count and serum ferritin at regular intervals post‐surgery	Grade B EL 2 (2+ to 2−)
•Consider the following frequency of monitoring of full blood count and ferritin levels: 3, 6 and 12 months in the first year and at least annually thereafter so that changes in status may be detected	GPP
Folate	
•Check serum folate levels at regular intervals post‐surgery	Grade B EL 2 (1+ to 2−)
•Consider the following frequency of monitoring of serum folate levels: 3, 6 and 12 months in the first year and at least annually thereafter so that changes in status may be detected	GPP
Vitamin B12	
•Check vitamin B12 levels at regular intervals following SG, RYGB and malabsorptive procedures such as BPD/DS	Grade B EL 2 (2++ to 2−)
•Consider the following frequency of monitoring of vitamin B12 levels: 3, 6 and 12 months in the first year and at least annually thereafter so that changes in status may be detected	GPP
Vitamin D, calcium and parathyroid hormone	
Vitamin D	
•Check serum 25‐hydroxyvitamin D levels at regular intervals post‐surgery	Grade B EL 2 (1+ to 3)
•Serum 25‐hydroxyvitamin D levels of 75 nmol/L or greater are considered sufficient.	Grade D EL 4
•Ensure total 25‐hydroxyvitamin D (D3 and D2) is measured if patient is on vitamin D2 supplements, e.g., ergocalciferol	GPP
•Consider the following frequency of monitoring of vitamin D levels: 3, 6 and 12 months in the first year and at least annually thereafter so that changes in status may be detected	GPP
Calcium	
•Check serum calcium levels at regular intervals	GPP
•Consider the following frequency of monitoring of serum calcium levels: 3, 6 and 12 months in the first year and at least annually thereafter so that changes in status may be detected	GPP
Parathyroid hormone	
•Check parathyroid hormone (to exclude primary hyperparathyroidism) if it has not been checked prior to surgery	GPP
Fat‐soluble vitamins A, E and K	
Vitamin A	
• Consider checking serum vitamin A levels if patient reports steatorrhoea or symptoms of vitamin A deficiency, for example, night blindness or protein malnutrition	Grade D EL 4 (2+ to 4)
•Check serum vitamin A levels at regular intervals following malabsorptive procedures such as BPD/DS	Grade B EL 2 (1+ to 2)
•Consider the following frequency of monitoring of serum vitamin A levels following malabsorptive procedures such as BPD/DS: every 3 months and then annually once levels are stable	GPP
Vitamin E	
•Check serum vitamin E levels at regular intervals following malabsorptive procedures such as BPD/DS	Grade B EL 2 (1+ to 2+)
•Consider monitoring of serum vitamin E levels at least annually following malabsorptive procedures such as BPD/DS	GPP
•Check serum vitamin E levels if unexplained anaemia or neuropathy	Grade D EL 4
Vitamin K	
•Check vitamin K1 and PIVKA‐II levels at regular intervals following malabsorptive procedures such as BPD/DS	Grade B EL 2 (1+ to 3)
•Consider monitoring of serum vitamin K1 and PIVKA levels at least annually following malabsorptive procedures such as BPD/DS	GPP
Trace minerals: zinc, copper, selenium and magnesium	
Zinc	
•Check serum/plasma zinc levels at regular intervals following SG, RYGB or BPD/DS	Grade B EL 2 (1+ to 3)
•Consider monitoring serum/plasma zinc levels at least annually following SG, RYGB or BPD/DS	GPP
•Check serum/plasma zinc levels if unexplained anaemia, hair loss or changes in taste acuity	GPP
Copper	
•Check serum copper levels at regular intervals following SG, RYGB or BPD/DS	Grade C EL 3 (2− to 3)
•Consider monitoring serum copper levels at least annually following SG, RYGB or BPD/DS	GPP
• serum copper levels if unexplained anaemia or poor wound healing	GPP
•Serum copper should be monitored in patients taking zinc supplements and vice versa	GPP
Selenium	
•Check serum selenium levels if there is chronic diarrhoea, metabolic bone disease, unexplained anaemia or unexplained cardiomyopathy	Grade D EL 4
•Check serum selenium levels at regular intervals following RYGB •Check serum selenium levels at regular intervals following malabsorptive procedures such as BPD/DS •Consider monitoring serum selenium levels at least annually following RYGB or malabsorptive procedures such as BPD/DS	Grade D EL 2 (2−) Grade C EL 2 (2+) GPP
Thiamine	
•If the patient presents with rapid weight loss, poor dietary intake, vomiting, alcohol abuse, oedema or symptoms of neuropathy, initiate treatment for thiamine deficiency immediately. Do not delay pending blood results	GPP
HbA_1c_, lipids	
•Monitor HbA1c in patients with preoperative diabetes	GPP
•Monitor lipids in patients with preoperative dyslipidaemia	GPP

Abbreviations: AGB, adjustable gastric band; BPD/DS, duodenal switch; EL, evidence level and depicts where the majority of evidence lies; GPP, good practice point; PIVKA‐II, protein induced by vitamin K absence or antagonism; RYGB, Roux‐en‐Y gastric bypass, SG, sleeve gastrectomy.

### 7.1 Urea and electrolytes, renal and liver function tests

Renal function should be monitored for all procedures. Initially following surgery, people may have difficulty in maintaining an adequate fluid intake and become dehydrated.^51^ Routine postoperative monitoring of liver function is also appropriate,^2^, ^84^ to document changes in NAFLD, as well as part of general safety monitoring for hypoalbuminaemia, which may indicate underlying infection and inflammation.

### 7.2 Haematinics

There is a high incidence of iron deficiency anaemia following bariatric surgery because of low dietary iron intake, reduced intestinal absorption and, for some women, menstruation.^15^, ^18^, ^19^, ^35^, ^36^, ^37^, ^41^, ^57^, ^58^, ^59^, ^60^, ^72^, ^73^, ^74^, ^85^, ^86^, ^87^, ^88^, ^89^, ^90^, ^91^, ^92^, ^93^, ^94^

Vitamin B12 absorption is adversely affected by SG, RYGB and BPD/DS as it requires an acidic environment and presence of intrinsic factor produced by the gastric parietal cells.^95^ Many people have about 2‐year stores of vitamin B12; therefore, deficiency may present several years after surgery.^15^, ^18^, ^19^, ^40^, ^41^, ^47^, ^57^, ^58^, ^59^, ^65^, ^73^, ^86^, ^87^, ^88^, ^89^, ^92^, ^93^, ^96^ Vitamin B12 deficiency impacts adversely on the haematopoietic and nervous systems and may result in megaloblastic anaemia and irreversible neuropathies.^95^ Vitamin B12 levels are not a good predictor of deficiency because methodological problems affect sensitivity and specificity.^97^ In view of this, methylmalonic acid (MMA) has been proposed as a better indicator, but this requires a sensitive plasma assay not routinely available in the United Kingdom.^98^, ^99^ If there is doubt about vitamin B12 deficiency, it is better to treat.^97^

Because folic acid is absorbed in the small bowel,^95^ absorption may be affected by RYGB or BPD/DS, but deficiency is also observed following SG so is more likely to be due to low dietary intake or non‐adherence with vitamin and mineral supplementation.^15^, ^18^, ^19^, ^32^, ^34^, ^40^, ^41^, ^47^, ^87^, ^100^, ^101^

It should be noted that megaloblastic and macrocytic anaemia, associated with vitamin B12 deficiency, can be masked by folic acid supplementation, high folate levels or iron deficiency.^95^ It is essential to assess all haematinics before recommending additional folic acid supplements.^97^, ^102^

### 7.3 Vitamin D, calcium and PTH

Vitamin D plays an important part in musculoskeletal health and is essential for calcium absorption and bone mineralization.^44^, ^48^, ^64^, ^103^, ^104^ Following bariatric surgery, the desirable serum 25‐hydroxyvitamin D (25OHD) levels to optimize bone health, prevent secondary hyperparathyroidism, improve bone mineral density and calcium balance, and decrease fracture risk, are not known.^44^, ^48^ Many guidelines recommend 25OHD levels of >75 nmol L^−1^.^2^, ^3^, ^4^, ^52^ However, Chaktoura suggested that these were based on expert opinion rather than robust evidence and proposed serum 25OHD levels of 50 nmol L^−1^ with a call for further research.^48^

Normal 25OHD levels accompanied by persistently elevated PTH and high serum calcium levels may be an indication of primary hyperparathyroidism hence the need to check PTH levels at baseline.^10^ All people are at risk of developing vitamin D deficiency following bariatric surgery,^44^ but those who have more malabsorptive procedures are at greater risk.^68^, ^69^, ^70^, ^71^, ^72^, ^73^, ^89^, ^94^ Therefore, serum calcium and 25OHD levels should be monitored following bariatric surgery. If vitamin D supplementation is adjusted, serum calcium and 25OHD levels should be rechecked.^105^

### 7.4 Fat‐soluble vitamins A, E and K

Vitamin A deficiency has been reported after RYGB and in adolescents.^15^, ^93^, ^106^, ^107^, ^108^ Although clinical problems rarely occur, consideration should be given to monitoring serum vitamin A levels if there are concerns such as deterioration in night vision, dry eyes or protein–energy malnutrition.^3^, ^4^, ^53^ Vitamin A deficiency is more common following BPD/DS.^68^, ^69^, ^70^, ^71^, ^72^, ^73^, ^74^ Consequently, after malabsorptive procedures such as BPD/DS procedures, there should be routine monitoring of serum vitamin A levels. Many primary and secondary care providers are unable to request vitamin A monitoring, and interpretation of levels should be treated with caution as they do not directly reflect the body's vitamin A pool.^109^

Vitamins E and K deficiencies have been reported following BPD/DS,^69^, ^70^, ^72^, ^73^, ^94^ but we found no reports after AGB, SG^32^ or RYGB.^93^ Vitamin E is normally assessed by serum α‐tocopherol, which is transported non‐specifically in lipoproteins. When considering vitamin E nutritional status, adjustment should therefore be made for serum lipid levels.^110^ Vitamin K levels should be monitored by measuring serum vitamin K1 and protein induced by vitamin K absence or antagonism (PIVKA‐II).^111^ This creates a dilemma as many centres are unable to request measurement of vitamin K, and coagulation screens are not a reliable indication of vitamin K status.^111^ Consequently, we recommend that postoperative care of people following malabsorptive procedures (BPD/DS) should remain with specialist centres lifelong with routine monitoring of serum vitamin E and K levels.

Following AGB, SG or RYGB, routine monitoring of serum vitamin E and K levels is not recommended but may be measured in people presenting with unexplained anaemia, neuropathy, bruising or uncontrolled bleeding.^2^, ^3^, ^4^, ^51^

### 7.5 Trace minerals: zinc, copper, selenium and magnesium

Zinc deficiency occasionally occurs following SG or RYGB and more commonly following BPD/DS.^31^, ^36^, ^37^, ^53^, ^58^, ^67^, ^69^, ^72^, ^73^, ^74^, ^89^, ^94^, ^101^, ^112^, ^113^ Factors may include length of time after surgery and limb length.^112^, ^113^ Zinc deficiency may present as poor wound healing, taste changes, glossitis and hair loss.^53^ Serum/plasma zinc levels should be monitored if there are unexplained symptoms including anaemia or changes in taste acuity and at least annually following SG, RYGB and BPD/DS.^2^, ^3^, ^4^

Copper deficiency may present following RYGB^37^, ^67^, ^114^, ^115^, ^116^, ^117^ and more commonly following BPD/DS.^72^, ^89^, ^112^ Symptoms include anaemia, leucopoenia, thrombocytopenia and neuromuscular abnormalities.^114^, ^115^, ^118^, ^119^, ^120^ Elhag et al.^19^ reported a fall in copper levels in adolescents who had SG. In addition, high‐dose zinc supplementation over time can cause copper deficiency and vice versa.^118^, ^119^, ^120^ Serum copper should be monitored following SG, RYGB and BPD/DS, in people who are on high doses of zinc and in people who present with unexplained anaemia and myeloneuropathy.^2^, ^3^, ^4^

Serum selenium levels are not commonly measured, but deficiency has been reported after SG, RYGB and BPD/DS.^67^, ^69^, ^73^, ^89^, ^107^ Given that there are reports of selenium deficiency, we recommend routine monitoring of serum selenium levels after SG, RYGB and BPD/DS or if there is chronic diarrhoea, metabolic bone disease, unexplained anaemia or unexplained cardiomyopathy.^2^, ^4^

There was insufficient evidence to recommend routine monitoring of magnesium; however, people with hypocalcaemia should be investigated for hypomagnesaemia and treated prior to calcium supplementation.^121^

### 7.6 Thiamine

People are at risk of developing thiamine deficiency if they experience prolonged vomiting, rapid weight loss, poor dietary intake, alcohol abuse, oedema or symptoms of neuropathy.^34^, ^122^, ^123^, ^124^, ^125^, ^126^, ^127^, ^128^ All healthcare professionals involved in the aftercare of bariatric surgery patients should be aware of the potential risk for severe thiamine deficiency (see Sections 8.9 and 9.6).

Symptoms of thiamine deficiency include ataxia, confusion and coma (Cerebral Beri Beri and Wernicke's encephalopathy), neuropathy and neuritis especially in lower limbs (Dry Beri Beri) or cardiac insufficiency with tachycardia and respiratory symptom (Wet Beri Beri).^95^, ^124^, ^125^ If thiamine deficiency is suspected, either because of risk factors or clinical symptoms, oral or intravenous treatment should be initiated immediately and not delayed pending tests results.

### 7.7 Dyslipidaemia

Treatment needs for dyslipidaemia should be reassessed after achievement of weight loss.^10^

## 8 VITAMIN AND MINERAL SUPPLEMENTATION

Because peoples' requirements and adherence may vary over time, we recommend that supplements should be reviewed regularly and adjusted accordingly. Details of the recommended vitamin and mineral supplements are shown in Table 4.

**TABLE 4:** Postoperative vitamin and mineral supplementation

Postoperative vitamin and mineral supplementation	Grade, evidence level (EL), (range of evidence)
Recommendations	
•Vitamin and mineral supplements should be reviewed regularly and adjusted accordingly	GPP
•A complete multivitamin and mineral supplement (containing thiamine, iron, selenium, zinc and copper) is recommended daily after all bariatric procedures	GPP
Iron	
•Following AGB, consider recommending a multivitamin and mineral supplement containing iron to people, especially adolescents, as oral dietary intake of iron may be low	GPP
•Following SG, RYGB or malabsorptive procedures such as BPD/DS, recommend that people take additional elemental iron	Grade B EL 2 (1+ to 2−)
•Consider starting with 200‐mg ferrous sulphate, 210‐mg ferrous fumarate or 300‐mg ferrous gluconate daily and twice daily in menstruating women and adjust depending on blood results	Grade B EL 2 (1+ to 2−)
•Consider advising people to take iron supplements with citrus fruits/drinks or vitamin C	GPP
•Consider advising people to take calcium and iron 2 h apart as one may inhibit absorption of the other	GPP
Folic acid	
•Advise people to take a complete multivitamin and mineral supplement providing 400‐ to 800‐μg folic acid per day	Grade D EL 4 (1+ to 4)
Vitamin B12	
•Following SG, RYGB or malabsorptive procedures such as BPD/DS, recommend routine supplementation with vitamin B12 intramuscular injections	Grade B level 2 (1+ to 2−)
•Following SG, RYGB or malabsorptive procedures such as BPD/DS, recommended frequency of vitamin B12 intramuscular injections is every 3 months	GPP
Calcium and vitamin D	
Vitamin D	
•Adjust vitamin D3 supplementation to maintain serum 25‐hydroxyvitamin D levels of 75 nmol L^−1^ or higher	Grade D EL 4 (2 to 4)
•Maintenance levels of between 2000 and 4000 IU oral vitamin D3 per day may be required following SG and RYGB and higher following malabsorptive procedures such as BPD/DS	Grade D EL 4 (2 to 4)
Calcium	
•Ensure good dietary calcium intake, recognizing that requirements may be higher in individuals who have SG, RYGB or malabsorptive procedures such as BPD/DS. If PTH is raised, despite adequate serum 25‐hydroxyvitamin D levels and calcium is normal then consider a combined vitamin D and calcium supplement	GPP
•To aid calcium absorption, advise that calcium taken as equally divided doses; calcium carbonate with food; calcium citrate with or without food	GPP
•Calcium citrate may be the preferred supplement for people at risk of developing kidney stones	GPP
Vitamins A, E and K	
Vitamin A	
•Following bariatric surgery, recommend that individuals take a complete multivitamin and mineral supplement containing U.K. government dietary recommendations for vitamin A	GPP
•Following RYGB, especially in people, consider that some may require additional routine oral vitamin A supplementation, especially if symptoms such as deterioration in night vision and dry eyes are present	Grade C EL 2 (1− to 4)
•Following malabsorptive procedures such as BPD/DS, recommend daily supplementation with additional oral vitamin A	Grade B EL 2 (1+ to 3)
•Following malabsorptive procedures such as BPD/DS, we suggest starting at 10 000 IU (3000 μg) oral vitamin A daily and adjust as necessary	GPP
Vitamin E	
•Following malabsorptive procedures such BPD/DS, recommend daily oral supplementation with additional vitamin E	Grade C EL 2 (1+ to 4)
•Following malabsorptive procedures such BPD/DS, we suggest starting with 100‐IU oral vitamin E daily and adjust as necessary	GPP
Vitamin K	
•Following malabsorptive procedures such BPD/DS, recommend daily oral supplementation with additional vitamin K	Grade C EL 2 (1+ to 4)
•Following malabsorptive procedures such BPD/DS, we suggest starting with 300‐μg oral vitamin K daily	GPP
Water‐miscible forms of fat‐soluble vitamins	
•Water‐miscible forms of fat‐soluble vitamins may improve absorption especially after malabsorptive procedures	Grade D EL 4
Zinc and copper	
•Recommend a multivitamin and mineral containing at least the government recommended daily allowance for zinc	Grade B EL 2
•Following RYGB and SG, the optimal level of zinc supplementation is not known; however, we recommend 15‐mg zinc oral daily, which may be contained within the multivitamin and mineral supplement	GPP
•Following malabsorptive procedures such BPD/DS, the optimal level of zinc supplementation is not known but will be higher than that for RYGB or SG. We recommend starting with at least 30‐mg oral zinc daily, which may be contained within the oral multivitamin and mineral supplement	Grade C EL 2
•Following RYGB, SG and BPD/DS, recommend complete multivitamin and mineral oral supplement containing 2‐mg copper	Grade D EL 4
Selenium	
•Recommend a complete multivitamin and mineral supplement containing selenium	Grade D EL 2 (2−)
•Following malabsorptive procedures such as BPD/DS, additional routine oral supplementation with selenium may be needed to prevent deficiency	Grade B EL 2 (1+ to 2−)
Thiamine	
•Recommend a complete multivitamin and mineral supplement containing at least government dietary recommendations for thiamine	Grade B EL 2
•Consider recommending oral thiamine or vitamin B co strong tablets for first 3‐ to 4‐month post‐surgery	GPP
•Prescribe oral thiamine 200–300 mg daily, vitamin B co strong 1 or 2 tablets, three times a day to people with symptoms such as dysphagia, vomiting, poor dietary intake or fast weight loss	Grade D EL 4
•Clinicians should be educated about the factors, which may predispose to thiamine deficiency and the importance of initiating immediate treatment	GPP
•People should be educated about the risks of potential thiamine deficiency and asked to seek early advice if they experience prolonged vomiting or poor dietary intake	GPP

Abbreviations: AGB, adjustable gastric band; BPD/DS, duodenal switch; EL, evidence level and depicts where the majority of evidence lies; GPP, good practice point; RYGB, Roux‐en‐Y gastric bypass; SG, sleeve gastrectomy.

### 8.1 Complete multivitamin and mineral supplements

Following all bariatric procedures, a complete multivitamin and mineral supplement (containing thiamine, iron, zinc, copper and selenium) is recommended.^2^, ^3^, ^4^ A minimum of 2 mg of copper and 15‐mg zinc per day is advised following SG, RYGB and BPD/DS (see Section 8.7). Care should be taken to check that the multivitamin and mineral supplement contains sufficient amounts of vitamins and minerals to counter the malabsorptive effects of bariatric surgery; however, additional supplements will be needed.

### 8.2 Iron

Following insertion of AGB, people should be able to meet their iron requirements by oral diet and a complete multivitamin and mineral supplement containing the recommended daily allowance of iron. A dietetic review may be needed to check nutritional adequacy of their diet.^129^

Following SG, RYGB and malabsorptive procedures such as BPD/DS, a complete multivitamin and mineral supplement may not prevent iron deficiency, and additional elemental iron is required.^2^, ^3^, ^4^, ^19^, ^34^, ^35^, ^74^, ^92^, ^130^ We suggest adding 200‐mg ferrous sulphate, 210‐mg ferrous fumarate or 300‐mg ferrous gluconate daily. However, this may not be sufficient to prevent anaemia.^35^, ^47^, ^74^, ^88^, ^89^ Women, of reproductive age and who are menstruating, have additional requirements of 50‐ to 100‐mg elemental iron daily (i.e., two 200‐mg ferrous sulphate or 210‐mg ferrous fumarate tablets daily).^53^, ^130^ Taking iron supplements alongside citrus fruits/drinks or vitamin C may aid absorption. Iron and calcium supplements should be taken 1–2 h apart to avoid affecting absorption of each.^3^, ^53^

### 8.3 Folic acid

Folic acid requirements for people who have undergone bariatric surgery are unknown. Complete multivitamin and minerals that contain less than 400 μg daily may not be sufficient to prevent deficiencies.^3^ There are increased requirements for preconceptual care, pregnancy and lactation (see Section 9.8).^11^, ^131^

### 8.4 Vitamin B12

Given the risk of developing vitamin B12 deficiency following SG, RYGB and BPD/DS (see Section 7.2), additional vitamin B12 supplementation is required for prevention. Untreated vitamin B12 deficiency may result in irreversible neuropathy or subacute combined degeneration of spinal cord, which may occur in the absence of megaloblastic anaemia.^95^, ^132^

Discussion continues as to the optimal dosage and route of supplementation.^133^, ^134^, ^135^ Some studies suggest that supplementation with high doses of oral vitamin B12 may prevent or reduce the development of deficiency.^34^, ^135^ Further research is needed, as deficiency may still occur in the presence of high oral doses.^34^, ^92^, ^136^ Adherence to oral supplementation is more difficult to determine.^34^, ^96^, ^133^, ^134^, ^135^ Given the serious consequences of vitamin B12 deficiency, we recommend routine supplementation with three monthly intramuscular vitamin B12 injections for SG, RYGB and malabsorptive procedures such as BPD/DS.^134^

### 8.5 Calcium and vitamin D

Vitamin D3 supplementation requirements to maintain optimal serum 25OHD levels greater than 75 nmol L^−1^ post‐bariatric surgery are higher than for a non‐bariatric surgery population.^3^, ^4^, ^10^, ^44^, ^48^, ^52^, ^62^, ^137^ Starting regimens of 2000–4000 IU of vitamin D3 per day are recommended to maintain serum 25OHD levels after surgery with adjustments being made dependent on results.^3^, ^48^, ^62^ With malabsorptive procedures such as BPD/DS, higher doses of vitamin D3 supplementation are likely to be needed.^52^, ^69^, ^70^, ^74^ Dietary intake of vitamin D and weight bearing activity should also be encouraged.^51^

Calcium absorption is adversely affected by bariatric surgery.^70^, ^138^ Several guidelines recommend additional calcium supplementation.^2^, ^3^, ^4^, ^9^, ^51^ However, the optimal calcium intake for people who have had bariatric surgery is not known. Dietary sources of calcium should be encouraged as it is more bioavailable than supplemental calcium and may have a protective role in the formation of kidney stones.^139^ Parrott et al. recommend 1200‐ to 1500‐mg calcium per day from food and supplements following AGB, SG and RYGB and 1800–2400 mg d^−1^ following BPD/DS.^3^

Discussion continues as to whether calcium citrate should be favoured over calcium carbonate. However, there have been no large RCTs comparing the two supplements for all bariatric procedures.^51^, ^103^, ^140^ Although calcium citrate may be more bioavailable and the preferred option for people at risk of kidney stones,^141^ calcium carbonate may be better tolerated.^3^ Parrott et al. advise taking calcium in divided doses, taking calcium carbonate with meals and calcium citrate with or without meals.^3^ Care should be taken to ensure that there is not excess calcium intake, and healthcare professionals should be mindful of the risk of kidney stones. Good hydration should be encouraged.^48^

### 8.6 Vitamins A, E and K

Following AGB and SG, people should be able to meet requirements for vitamins A, E and K through their oral diet and a complete multivitamin and mineral supplement. Given that lower levels of vitamin A or deficiency has been reported after RYGB,^93^, ^107^, ^108^ some people may require additional vitamin A supplementation. Whilst we did not find sufficient evidence to recommend routine additional supplementation beyond the complete multivitamin and mineral supplement, this may be identified through monitoring (see Section 7.4).

The optimum level of supplementation to prevent vitamin A deficiency following BPD/DS is not known. There are reports of vitamin A deficiencies developing despite supplementation of 5000–25 000 IU d^−1^.^71^, ^89^, ^94^ For BPD/DS with 100‐cm common channel, Homan et al.^74^ recommended starting vitamin A supplementation with 50 000 IU week^−1^, checking blood levels after 3 months and adjusting the dosage if necessary. Homan et al.^74^ also estimated that an oral dose of 7500–15 000 IU d^−1^ (solubilized vitamin A) is needed to prevent vitamin A deficiency after BPD/DS, while Topart et al.^89^ suggested that vitamin A 50 000 IU d^−1^ in tablet form was needed to prevent deficiency. Homan et al. noted that this difference may be due to differences in formulation, as solubilized forms may be better absorbed.^74^ We recommend starting with 10 000 IU d^−1^ vitamin A and adjusting depending on blood results to avoid over‐supplementation.^51^

For prevention of vitamin E deficiency has been found post BPD/DS, Slater et al. supplemented with 60 IU vitamin E per day although a small number of participants still presented with vitamin E deficiency at 4 years.^70^ We suggest starting with 100 IU vitamin E per day following BPD/DS and adjusting as required.

The optimal level of vitamin K supplementation to prevent deficiencies after malabsorptive procedures is not known. Although, Aills et al. suggest 300‐μg vitamin K daily.^51^

Water‐miscible forms of fat‐soluble vitamins may improve absorption especially after malabsorptive procedures.^2^, ^3^, ^4^, ^51^

### 8.7 Zinc and copper

The complete multivitamin and mineral supplement may not contain sufficient zinc to prevent deficiency following SG, RYGB or BPD/DS (see Section 7.5). Although the optimum level of zinc supplementation is not known, we suggest starting with 15 mg d^−1^ zinc, which may be contained within the multivitamin and mineral supplement. After BPD/DS, Topart et al.^89^ suggested starting with 30 mg d^−1^ zinc, whilst Homan et al.^74^ suggested 100 mg d^−1^ zinc is needed to prevent deficiency. Given that zinc and copper have an inverse relationship for absorption, we recommend starting with 30 mg d^−1^ zinc after BPD/DS.

We recommend that all people have a complete vitamin and mineral supplement that provides 2 mg d^−1^ copper.^67^, ^117^ People who have had long limbed gastric bypass or BPD/DS may have additional requirements.^74^, ^89^, ^112^

If additional zinc supplements are given, monitor both zinc and copper levels as normally a ratio of 8–15 mg of zinc for each 1‐mg copper should be maintained to avoid zinc‐induced copper deficiency.^3^, ^4^ The current complete multivitamin and mineral available on prescription (Forceval) in the United Kingdom contains 2‐mg copper and 15‐mg zinc, and doubling up on the dosage may be sufficient in some cases to meet the additional requirements.^112^

### 8.8 Selenium

Although the evidence is limited, a complete multivitamin and mineral supplement, which contains selenium, should be recommended after bariatric surgery. Additional selenium may be needed by some people following RYGB and especially following BPD/DS.^69^, ^73^, ^89^, ^107^ Over‐the‐counter preparations or Brazil nuts may also be used to supplement selenium.

### 8.9 Thiamine

People are at high risk of developing thiamine deficiency post‐bariatric surgery (see Section 7.6). Garg et al. reported that people who saw a dietitian had more favourable thiamine levels at 3‐month post‐surgery.^126^

The complete multivitamin and mineral supplement containing thiamine may not be sufficient to prevent deficiency. Parrott et al. recommend that all people take 12 mg d^−1^ thiamine and preferably 50‐mg dose of thiamine once or twice a day from a vitamin B‐complex supplement.^3^ We were unable to find sufficient evidence to support this; however, we recognize that further research and evidence are needed in this important area. For people in which there is concern that a complete multivitamin and mineral supplement containing thiamine is not sufficient, consideration should be given to prescribing additional thiamine or a vitamin B‐complex supplement for the first three to four postoperative months.

If people present with prolonged vomiting, dysphagia, poor nutritional intake, inability to tolerate vitamin and mineral supplements, high alcohol intake or fast weight loss, additional thiamine supplementation should be administered immediately to prevent the development of Wernicke's encephalopathy.^122^, ^124^, ^125^, ^126^, ^127^, ^128^ Consideration should be given to admission and immediate parenteral replacement with thiamine in people where thiamine deficiency is suspected; see Sections 7.6 and 9.6.

Given the severe consequences that arise from untreated or late treatment of thiamine deficiency, we recommend that people are educated about potential risks of thiamine deficiency and to seek early advice. Clinicians in both primary and secondary care need education on the factors that may predispose to thiamine deficiency and the importance of initiating immediate treatment.

## 9 ABNORMAL TEST RESULTS AND CLINICAL PROBLEMS

Detailed recommendations are listed in Table 5.

**TABLE 5:** Abnormal test results, clinical problems, pregnancy and adolescents

Abnormal test results, clinical problems, pregnancy and adolescents	
Recommendations	Grade, evidence level, range of evidence
Protein malnutrition/protein energy malnutrition/oedema	
•If people present with signs/symptoms of protein malnutrition/protein energy malnutrition/oedema, investigate potential causes and refer back to bariatric centre	GPP
Anaemia	
Iron deficiency anaemia	
•Sources of blood loss should be considered, investigated and excluded in individuals who present with iron deficiency anaemia	Grade D EL4
•For people over 12 years old and pregnant women diagnosed with iron deficiency anaemia, treat iron deficiency following NICE CKS Anaemia—iron deficiency	Grade D EL 4
Vitamin B12 deficiency	
•Treat vitamin B12 deficiency immediately using NICE CKS: Anaemia—B12 and folate deficiency. Do not give folic acid first as it may mask underlying vitamin B12 deficiency and precipitate subacute combined degeneration of the spinal cord	Grade D EL 4
•For people with neurological involvement, NICE recommend administering hydroxocobalamin 1 mg intramuscularly on alternate days until there is no further improvement, then administer hydroxocobalamin 1 mg intramuscularly every 2 months	Grade D EL 4
•For people with no neurological involvement, NICE recommend administering hydroxocobalamin 1 mg intramuscularly three times a week for 2 weeks	Grade D EL 4
•After treatment of vitamin B12 deficiency, provide maintenance treatment with 1 mg intramuscularly every 2–3 months lifelong	Grade D EL 4
•Seek urgent specialist advice from neurologist and haematologist, if there is possible neurological involvement, such as unexplained sensory and/or motor and gait symptoms	GPP
Folic acid deficiency	
•Check and treat for vitamin B12 deficiency, before initiating folic acid treatment to avoid precipitation of subacute combined degeneration of the spinal cord	Grade D EL 4
•Treat folic acid deficiency using NICE CKS: Anaemia—B12 and folate deficiency. Folic acid 5 mg orally daily for a minimum of 4 months is recommended and further investigations if there is suspicion of malabsorption	Grade D EL 4
Unexplained anaemia/fatigue	
•For unexplained causes of anaemia or fatigue, investigate for other nutritional deficiencies including protein, zinc, copper and selenium	GPP
Low vitamin D levels	
•In absence of local adult guidelines for vitamin D replacement, refer to National Osteoporosis Society guidance: Vitamin D and Bone Health: A Practical Clinical Guideline for Patient Management	Grade D EL 4
•In absence of local children and young people guidelines for vitamin D replacement, refer to National Osteoporosis Society guidance: A Practical Clinical Guideline for Patient Management in Children and Young People or a paediatrician	Grade D EL 4
•If the person remains vitamin D deficient despite treatment, refer to a secondary care specialist	GPP
•In people with severe vitamin D deficiency, high dose vitamin D injections might be required, which should be given following specialist consultation, in people with known history/high risk of hypercalcaemia, e.g., people with kidney stones, sarcoidosis, renal impairment and atrial fibrillation	GPP
Vitamin A deficiency/disturbances in night vision/xerophthalmia	
•In adults, treat vitamin A deficiency, with 10 000‐ to 25 000‐IU oral vitamin A daily for 1–2 weeks for clinical improvement. Recheck vitamin A levels at 3 months	Grade D EL 4
•For vitamin A deficiency that does not respond to treatment, refer to specialist for assessment and consideration of intramuscular vitamin A injections	GPP
•In adolescents, with vitamin A deficiency, refer for specialist support	GPP
Vitamin E and vitamin K deficiency	
•Treat vitamin E deficiency with oral vitamin E 100–400 IU d^−1^. Recheck levels after 3 months	Grade D EL 4
•For vitamin E deficiency that does not respond to treatment, refer to specialist for assessment and consideration of intramuscular injections	GPP
•When considering vitamin E nutritional status, adjustment should be made for serum lipids	Grade D EL 4
•For vitamin K deficiency, treat with 1‐ to 2‐mg oral vitamin K daily (Ketovite tablets, menadiol sodium phosphate or phytomenadione). Recheck levels after 3 months. For those on anticoagulants such as warfarin or for vitamin K deficiency that does not respond to treatment, refer to specialist for assessment	GPP
Neurological symptoms/Wernicke's encephalopathy	
In patients who present with neurological symptoms:	
•Treat for thiamine deficiency (see section prolonged vomiting/dysphagia/poor oral intake/risk of thiamine deficiency)	Grade D EL 4
•Check for vitamin B12, copper and vitamin E deficiencies and treat	GPP
•Refer to neurologist and haematologist	GPP
Zinc and copper deficiency	
•If both zinc and copper low, consider prescribing two Forceval daily for 3 months and recheck levels	GPP
•Check both zinc and copper levels when considering zinc or copper replacement •With mild zinc or copper deficiency, consider giving two Forceval daily and recheck levels after 3 months	Grade D EL 4 GPP
•With severe zinc deficiency and normal or borderline copper levels, treat with high dose zinc supplement for 3 months and recheck levels. If no improvement or copper levels fall, refer for specialist advice	GPP
•With severe copper deficiency, refer for specialist advice	GPP
•When giving additional zinc and copper, maintain a ratio of 8‐ to 15‐mg zinc to 1‐mg copper. Close monitoring is required if higher zinc or copper doses are indicated because each affects the absorption of the other. If necessary, ask for expert advice	Grade D EL 4
Prolonged vomiting/dysphagia/poor oral intake/risk of thiamine deficiency	
•If people present with prolonged vomiting or dysphagia, refer back to the bariatric centre for investigation	GPP
•People, who present with prolonged vomiting or dysphagia, are at risk of thiamine deficiency. Give additional thiamine and vitamin B co strong immediately (thiamine 200–300 mg daily, vitamin B co strong 1 or 2 tablets, three times a day)	Grade D EL 4
•For those unable to tolerate thiamine orally or with clinical suspicion of acute deficiency intravenous thiamine should be given	Grade D EL 4
Pregnancy	
•Women are advised to avoid pregnancy for the first 12–18 months following surgery to allow weight stabilization and a varied nutritious diet	Grade D EL 4
•Women with a BMI < 29.9 kg m^2^, planning for pregnancy, should take an additional 400 micrograms/day folic acid prior to conception until the 12th week of pregnancy	Grade D EL 4
•Women with type 2 diabetes mellitus or a BMI > 30 kg m^2^ should take 5‐mg folic acid until the 12th week of pregnancy. Check for vitamin B12 deficiency before starting	Grade D EL 4
•Refer to specialist antenatal care	Grade D EL 4
•Replace vitamin A in supplements from retinol to beta carotene form or take preconception or pregnancy specific vitamin and mineral supplement	Grade D EL 4
•Pregnant women, following bariatric surgery, should undergo nutritional screening during each trimester. This should include ferritin, folate, vitamin B12, calcium, vitamin D, vitamin A	Grade D EL 4
•Pregnant women, following bariatric surgery, especially those who have had long‐limbed bypass or BPD/DS procedures, may be at risk of low vitamins E and K levels. These should be monitored during pregnancy if clinically indicated	Grade D EL 4
•A more frequent review with the specialist bariatric dietitian may be required	Grade D EL 4
•Reference ranges change in pregnancy. Please refer to perinatal reference ranges when checking blood results http://perinatology.com/Reference/Reference Ranges/Reference for Serum.htm	GPP
Adolescents	
•Adolescents who have undergone bariatric surgery should be monitored for dietary adherence and nutritional assessment on a regular basis due to changes in body composition, growth and sexual development	GPP
Malabsorptive procedures	
•Individuals who have malabsorptive procedures have a higher prevalence of post‐surgery nutritional deficiencies and care should remain with the specialist centre	GPP
•For OAGB/MGB with BP limb length of 150 cm or less, follow RYGB nutritional recommendations	GPP
•For OAGB/MGB with BP limb length of greater than 150 cm or SADIs, follow BPD/DS nutritional recommendations	GPP

Abbreviations: BPD/DS, duodenal switch; CKS, clinical knowledge summary; EL, evidence level and depicts where the majority of evidence lies; GPP, good practice point; OAGB/MGB, one anastomosis gastric bypass/mini gastric bypass; RYGB, Roux‐en‐Y gastric bypass; SADIs, single anastomosis duodenal ileal bypass with sleeve gastrectomy.

### 9.1 Protein malnutrition/protein–energy malnutrition/oedema

This can present several years following bariatric surgery. Causes include poor dietary protein intake as well as malabsorption. People who have difficulty progressing their diet because of a stricture, overtight band or food intolerances are at risk in addition to those with protein malabsorption.^69^, ^71^, ^89^, ^94^, ^142^

Oedema is an important indicator of protein energy malnutrition and may mask muscle wasting and weight loss. Whilst it is necessary to exclude the many other causes of oedema, people should also be referred back to the bariatric centre for further investigation.

### 9.2 Anaemia

#### 9.2.1 Iron deficiency anaemia

Iron deficiency anaemia may be dietary in origin, with oral diet and iron supplements being insufficient to meet the needs of people following bariatric surgery (see Section 7.2). Sources of blood loss, both related and unrelated to bariatric surgery, should also be considered, investigated and excluded.^143^

If there is a low haemoglobin and low mean cell volume (MCV), serum ferritin levels should be measured. Levels of serum ferritin less than 15 μg L^−1^ confirm iron deficiency anaemia.^143^ Acute and chronic inflammatory conditions, liver disease and malignant disease may result in increased ferritin levels independent of iron status; therefore, people with chronic inflammation and a ferritin concentration of 50 μg L^−1^ or higher could still be iron deficient. If inflammation is thought to be affecting the ferritin levels, other markers of inflammation such as C‐reactive protein or measures of iron status such as total iron binding capacity should be considered. If there is doubt about the tests to be requested or interpretation of results, specialist advice should be sought.^143^

#### 9.2.2 Vitamin B12 and folate

The most common causes of megaloblastic, macrocytic anaemia are vitamin B12 and folate deficiency,^132^ and folate supplementation may mask severe vitamin B12 depletion (see Section 7.2). It is essential that vitamin B12 deficiency is treated immediately before initiating additional folic acid (see Section 7.2).

In vitamin B12 deficiency with possible neurological involvement, such as unexplained sensory and/or motor and gait symptoms, vitamin B12 deficiency should be treated immediately and urgent specialist advice sought from a neurologist and haematologist.^132^ Hydroxocobalamin 1 mg intramuscularly should be administered on alternate days until there is no further improvement, then hydroxocobalamin 1 mg intramuscularly administered every 2 months.^132^ For people with vitamin B12 deficiency and no neurological involvement, hydroxocobalamin 1 mg intramuscularly should be administered three times a week for 2 weeks, followed by maintenance treatment with 1 mg intramuscularly every 2–3 months for life.^132^

Folic acid deficiency may indicate non‐adherence with the daily multivitamin and mineral supplement or malabsorption. Some medications, such as anticonvulsants, sulfasalazine and methotrexate, may affect folic acid levels.^102^ For treatment of folic acid deficiency (after excluding vitamin B12 deficiency), oral folic acid 5 mg daily should be given for a minimum of 4 months.^132^

#### 9.2.3 Unexplained anaemia

Unexplained anaemia or fatigue may be a symptom of other nutritional deficiencies including protein, zinc, copper and selenium so levels of these should all be checked.^2^, ^3^, ^4^, ^114^, ^115^, ^117^, ^144^

### 9.3 Low vitamin D levels

Although low serum 25OHD levels are not a barrier to bariatric surgery, vitamin D insufficiency or deficiency should be treated preoperatively especially where the surgical procedure is likely to result in reductions in serum 25OHD levels.^3^, ^44^, ^48^, ^52^ Following surgery, if people present with vitamin D insufficiency/deficiency, adherence with the recommended supplements should be checked. However, for many people, despite good adherence, additional supplementation with vitamin D and higher maintenance doses may be required.^3^, ^44^, ^48^, ^62^, ^63^

Vitamin D deficiency requires correction with loading doses of vitamin D.^3^, ^48^ Many healthcare professionals have access to local guidelines/protocols for treatment of vitamin D deficiency. In the absence of these, the Royal Osteoporosis Society (ROS) gives recommendations for replacement.^105^ For adolescents, refer to ROS paediatric guidelines.^145^ For those people who remain vitamin D deficient or need a more aggressive approach, a referral to a secondary care specialist is recommended.

### 9.4 Vitamin A deficiency

Vitamin A deficiency can lead to visual problems such as xerophthalmia and loss of night vision^106^, ^108^ and reduced male fertility and may also result in foetal abnormalities.^95^, ^146^ Vitamin A levels should be measured if there are clinical concerns, and if appropriate, a referral to an ophthalmologist should be considered. For treatment of vitamin A deficiency in adults, oral supplementation with vitamin A, 10 000–25 000 IU d^−1^ for 1–2 weeks for clinical improvement is recommended.^3^, ^72^, ^84^ Higher doses, including intramuscular injections, may be needed if the person is experiencing night blindness.^3^, ^4^, ^147^ The levels should be rechecked at 3 months.^72^ For vitamin A deficiency that does not respond to oral supplementation, an onward referral to a specialist is needed as intramuscular injections may be required.

Silva et al. highlight that there are no guidelines for vitamin A replacement in adolescents.^16^ We recommend seeking specialist advice.

### 9.5 Vitamin E and vitamin K deficiency

Symptoms of vitamin E deficiency include peripheral neuropathy, muscle weakness and ataxia.^84^ Oral vitamin E 100–400 IU d^−1^ has been recommended for maintenance of vitamin E levels recognizing more may be needed for repletion,^3^, ^51^ while Bays et al. suggested 400‐ to 800‐IU vitamin E.^53^ Serum levels should be monitored and treatment continued until serum levels reach normal range.^3^, ^53^, ^84^ For vitamin E deficiency that does not respond to oral supplementation, refer onto a specialist as intramuscular injections may be needed.

Large vitamin E doses can result in over‐replacement and exacerbate vitamin K deficiency and therefore affect blood coagulation, so care should be taken.^95^ Furthermore, assessment of vitamin K should be performed when there is established fat‐soluble vitamin deficiency with hepatopathy, coagulopathy or osteoporosis.^2^, ^4^, ^148^ For vitamin K deficiency, Ketovite tablets contain acetomenaphthone (vitamin K) 500 μg per tablet and may be used to treat vitamin K deficiency although menadiol sodium phosphate or phytomenadione may be taken orally.^149^ An oral dose of 1–2 mg d^−1^ is recommended.^3^ Seek further advice from a haematologist if people are taking anticoagulants such as warfarin. Onward referral is needed if vitamin K deficiency does not respond to treatment.

### 9.6 Deficiencies associated with neurological symptoms/Wernicke's encephalopathy

Myeloneuropathy may have a number of causes including deficiencies of vitamin B12, thiamine, copper or vitamin E. Beri Beri and Wernicke's encephalopathy are severe complications caused by thiamine deficiency.^95^ All these conditions may occur after bariatric surgery.^122^, ^124^, ^125^, ^127^, ^128^, ^150^

In people at risk of thiamine deficiency or with clinical suspicion of acute deficiency (see Sections 7.6 and 8.9), additional thiamine and vitamin B compound strong should be given immediately, i.e., oral thiamine 200–300 mg d^−1^ and vitamin B compound strong 1 or 2 tablets three times a day, or full dose daily intravenous vitamin B preparation, if necessary for those unable to tolerate thiamine orally.^125^, ^151^, ^152^ Oral or intravenous glucose must not be given to people at risk of or with suspected thiamine deficiency as it can precipitate Wernicke–Korsakoff syndrome.^122^, ^150^ All healthcare professionals, including emergency department staff, need to be aware of this preventable complication and its management. Note that prolonged vomiting or dysphagia is not normal and should always be investigated. A referral back to the bariatric centre is recommended.

Vitamin B12, copper and vitamin E levels should be assessed and any deficiencies corrected (see Sections 9.2.2, 9.5 and 9.7). People with neurological symptoms should be referred to a neurologist.

### 9.7 Zinc and copper deficiency

For borderline low zinc or copper levels, blood tests should be repeated at 3 months as levels may fluctuate. In these cases, levels may respond to two capsules a day of complete multivitamin and mineral supplement available on prescription (Forceval). Although normally zinc/copper ratio should be maintained when giving additional supplements, in the case of deficiency of one of these micronutrients and a normal level of the other, high doses of either one may be given providing the levels of both continue to be monitored. Where there is doubt, seek specialist advice.

### 9.8 Pregnancy

Women are often advised to avoid pregnancy for the first 12–18 months following bariatric surgery.^2^, ^4^, ^11^, ^131^ The evidence base for this recommendation is limited; however, this helps to ensure that the woman has reached weight stability is able to eat a varied nutritious diet and enables appropriate planning of pregnancy and associated care.^11^

Preconception care should be discussed with women who plan to become pregnant. All women, planning for pregnancy, should take additional folic acid to reduce the risk of foetal neural tube defects. Prior to conception and until the 12th week of pregnancy, 400 μg d^−1^ folic acid is recommended. However, in women with obesity or diabetes, the recommendation is 5 mg d^−1^ folic acid.^11^, ^131^, ^153^, ^154^ Vitamin B12 should be measured during the preconception period before additional folic acid supplements are given.

A systematic review by Jans et al. concluded that the evidence on micronutrient deficiencies in pregnant and postpartum women after bariatric surgery and subsequent adverse neonatal outcomes was weak and inconclusive and called for larger prospective cohort studies to be undertaken.^146^ In a retrospective study of 73 pregnancies in a single centre, Yau et al. did not find an increase in nutritional deficiencies in women post‐bariatric surgery who had a shorter time from surgery to conception.^155^ In other studies, the incidence of vitamin A deficiency has been seen to increase over pregnancy.^146^, ^156^, ^157^ The incidence of vitamin K deficiency has also been found to increase with the long‐limbed gastric bypass and BPD/DS during pregnancy.^158^ Vitamin K deficiency may be related to neonatal intracranial bleedings and birth defects. The frequency of nutritional monitoring should therefore be increased during pregnancy.^11^, ^131^ Reference values for levels may change over pregnancy, and it is useful to check results against sources such as those provided by perinatology.^159^

Women, as part of preconception care, are advised to avoid vitamin and mineral preparations, which contain vitamin A in the retinol form, in the first 12 weeks of pregnancy.^160^ However, this may leave them at risk of developing vitamin A deficiency.^146^, ^156^ Supplements containing retinol may increase the teratogenic risk especially in the first trimester.^95^ There are a number of vitamin and mineral supplements containing no vitamin A, which are specifically aimed at preconception and pregnancy. Supplements containing vitamin A in the beta carotene and not retinol form may be continued in pregnancy. It is important to check this; for example, the capsule form of the multivitamin and mineral supplement available on prescription is appropriate, but the soluble form is not.

Women, who become pregnant post‐bariatric surgery, should be treated as a specialist obstetric population with specific needs.^11^, ^131^ This includes access to specialist dietetic support and close monitoring of nutrition. They should undergo nutritional screening every trimester.^11^, ^131^ This should include ferritin, folate, vitamin B12, calcium, vitamin D and vitamin A.^11^ Those who have had long limbed bypass or BPD/DS procedures may be at risk of low vitamins A, E and K levels and so these should be monitored if clinically indicated.^146^ A more frequent review with the specialist bariatric dietitian may be required.^11^, ^131^ It should be noted that for women, who have had a gastric band, decisions around band management should be made in clinical context as there are RCTs to define optimum management of the gastric band inflation during pregnancy.^4^, ^161^

### 9.9 Adolescents

A number of studies, whilst giving favourable outcomes in adolescents undergoing bariatric surgery, reported both preoperative and postoperative nutritional deficiencies.^15^, ^16^, ^17^, ^18^, ^19^, ^162^ Adherence to nutritional supplements and engagement with postoperative follow‐up is a challenge in all age groups; however, adolescents are a vulnerable group and need regular monitoring and support through growth and sexual development.^12^, ^13^, ^14^, ^15^, ^16^, ^17^, ^18^, ^19^

### 9.10 Nutritional care of people who have had malabsorptive procedures

Malabsorptive procedures such as BPD/DS are associated with higher prevalence of post‐surgery nutritional deficiencies including fat‐soluble vitamins and trace minerals. The management of these including prevention and correction of deficiencies can be difficult. Healthcare professionals, especially in primary care, may be unable to request specific blood tests. In addition, people may not be able to access a full range of supplements. Unlicensed products may be needed for the correction of deficiencies. We recommend that individuals who have malabsorptive procedures remain under the care of specialist centres.

### 9.11 Newer procedures (OAGB/MGB and SADIs)

It is recognized that there is a growing interest in OAGB/MGB and SADIs and that, as yet, these are not covered robustly by clinical practice guidelines. Until such data emerge, it may be hypothesized that requirements for nutritional supplements in people undergoing OAGB/MGB with BP limb of 150 cm would be at least that of people undergoing RYGB, if not more, and that nutritional requirements for people undergoing OAGB/MGB with BP limb greater than 150 cm and SADIS would be at least that of people undergoing the traditional BPD/DS.^163^, ^164^, ^165^, ^166^ We therefore recommend that healthcare professionals follow RYGB nutritional recommendations for postoperative care after OAGB/MGB with BP limb length of 150 cm and BPD/DS recommendations for people after OAGB/MGB with BP limb length of greater than 150 cm or SADIs.

## 10 DISCUSSION

Clinical guidelines can make a vitally important contribution to patient safety, and a number of guidelines for the nutritional care of people undergoing bariatric surgery have been published over the last few years.^1^, ^3^, ^4^, ^8^, ^9^, ^10^, ^11^ However, not all have been specifically related to micronutrition, had dietetic involvement or detail on recommendations for the nutritional management of pregnancy post‐surgery. No previous nutritional guidelines for people undergoing bariatric surgery have included either primary care or patient representatives. Previous guidance aimed at the management of adolescents undergoing bariatric surgery has only included general nutritional recommendations,^12^, ^13^, ^14^ despite this vulnerable group being at high risk of developing nutritional deficiencies.^15^, ^16^, ^17^, ^18^, ^19^

Here, comprehensive, up‐to‐date guidelines have been developed using robust methodology that include graded recommendations for preoperative and postoperative nutritional monitoring, vitamin and mineral supplementation and correction of deficiencies for people undergoing bariatric surgery. Importantly, nutritional guidance for the care of adolescents and pregnancy post‐surgery has also been included, and the guidance development group was composed of a multidisciplinary team from primary and secondary care as well as patient representation. Thus, ensuring that all professions involved in preparing people for surgery and providing aftercare, as well as patient views and needs, were properly considered. Although these guidelines are aimed at improving practice in the U.K. setting and take account of the current model of healthcare in the United Kingdom, they are likely to be applicable to other healthcare settings. However, we have addressed issues specifically related to the U.K. healthcare system where possible; for example, both specialist and non‐specialist care providers may not always able to access all the suggested nutritional blood tests such as vitamin A and vitamin K. Correction of some nutritional deficiencies, especially fat‐soluble vitamins, can be a challenge as the use of off license medications may be required. In addition, it is of concern that in some parts of the United Kingdom, people are not given access to prescribed vitamin and mineral supplements, particularly for those undergoing malabsorptive procedures. These people will have higher requirements, but there is no easy access to supplements containing larger amounts of vitamins A, E and K. This is a potentially serious issue that must be addressed. Accessibility and affordability were amongst the factors affecting adherence to vitamin and mineral supplements after bariatric surgery in the United Kingdom.^167^

The main limitations of these guidelines are related to the current evidence base. There have been a very limited number of RCTs undertaken in bariatric surgery and nutrition.^34^, ^68^, ^101^, ^130^, ^135^, ^168^ There is also a lack of evidence for optimal nutritional supplementation after bariatric surgery, which is complicated by the fact that requirements will vary according to the surgical procedure, size of gastric pouch and length of common channel. Because of this lack of good quality evidence and robust studies in the area, the guideline groups were required to make a number of recommendations based on good practice and the expert opinion of the group (good practice points).

## 11 CONCLUSION

Preoperative dietetic assessment and biochemical monitoring are vital to ensure that people are able to meet nutritional needs after bariatric surgery and any nutritional deficiencies are identified and corrected.^3^ Long‐term biochemical monitoring is important for the detection and treatment of emergent postoperative nutritional deficiencies.^1^, ^2^, ^3^, ^4^, ^8^, ^9^, ^10^, ^11^, ^83^ Annual reviews enable nutritional supplements to be reviewed and adjusted. We have systematically reviewed the current evidence base for the nutritional care of patients undergoing bariatric surgery and report our consensus guidelines with graded recommendations. These guidelines have the potential to improve clinical practice and safety for people undergoing bariatric surgery and should be considered for adoption by healthcare organizations. They will be updated regularly as new evidence emerges.

## CONFLICT OF INTEREST

The guideline development group received no funding. The British Obesity and Metabolic Surgery Society funded the open‐access publication fees for this paper. MO, HP, JP, RW, JM, NW, DT, KC, JD, MS, AB, IM and JB have nothing to disclose. RB received funding to the university for trial participation from Novo Nordisk and personal fees from Noro Nordisk, GSK, and International Medicine Press. KM received honoraria from Medtronic Inc, Ethicon Inc, Gore Inc and various NHS Trusts for educational activities and mentoring colleagues through One Anastomosis Gastric Bypass procedure. CH received personal fees from Novo Nordisk, personal fees from Ethicon and personal fees from Napp. No authors declared any potential conflicts with industry relevant to these guidelines.

## Supporting information

Table S1 Search Strategy
Table S2 Quality Assessment Tools
Table S3: Full paper excluded studies with reasons for exclusions
