# Supplementary material for: British Obesity and Metabolic Surgery Society Guidelines on perioperative and postoperative biochemical monitoring and micronutrient replacement for patients undergoing bariatric surgery—2020 update
Source: Obes Rev. 2020 Aug 2;21(11):e13087. doi: 10.1111/obr.13087 (PMC7583474; doi:10.1111/obr.13087)
Supplement: Supplementary file 1 — Table S1 Search Strategy Table S2 Quality Assessment Tools Table S3: Full paper excluded studies with reasons for exclusions [file OBR-21-e13087-s001.pdf]

British Obesity and Metabolic Surgery Society Guidelines on peri-operative and postoperative biochemical monitoring and micronutrient replacement for patients undergoing bariatric surgery - 2020 Update

Supplementary information

Authors

Ms Mary O'Kane, Consultant Dietitian, Leeds Teaching Hospitals NHS Trust, Dietetic Department, The General Infirmary at Leeds, Great George Street, Calverley Street, Leeds, LS1 3EX

Dr Helen Parretti, Clinical Senior Lecturer in Primary Care, Norwich Medical School, University of East Anglia, Norwich, NR4 7TJ, UK

Professor Jonathan Pinkney, Professor of Endocrinology and Diabetes, Peninsula Schools of Medicine and Dentistry, Honorary Consultant Plymouth Hospitals NHS Trust

Mr Richard Welbourn, Consultant Upper GI and Bariatric Surgeon, Musgrove Park Hospital, Taunton TA1 5DA and Somerset NHS Foundation Trust

Dr Carly Hughes Fakenham Weight Management Service, Fakenham Medical Practice Norfolk and University of East Anglia; Norwich Medical School, University of East Anglia, Norwich, NR4 7TJ, UK

Dr Jessica Mok, Bariatric Surgery Clinical Research Fellow, Centre for Obesity Research, University College London

Nerissa Walker, Assistant Professor in Dietetics, School of Biosciences, Sutton Bonington Campus. University of Nottingham, LE12 5RD

Dr Denise Thomas, DClinPrac. RD, Specialist Dietitian, Portsmouth Hospitals NHS Trust

Jennifer Devin – Specialist Weight Management Service Lead/ Dietitian, Betsi Cadwaladr University Health Board

Dr Karen Coulman, HEE/NIHR ICA Clinical Lecturer. Population Health Sciences, Bristol Medical School. University of Bristol, 1-5 Whiteladies Road, Bristol BS8 1NU and Honorary Bariatric Surgery Dietitian, North Bristol NHS Trust

Gail Pinnock, Freelance bariatric surgery dietitian

Prof Rachel L Batterham, [a]Centre for Obesity Research, Rayne Institute, Department of Medicine, University College London, London WC1E 6JF, United Kingdom (UK).[b]University College London Hospital (UCLH) Bariatric Centre for Weight Management and Metabolic Surgery, UCLH, Ground Floor West Wing, 250 Euston Road, London NW1 2PG, United Kingdom (UK).[c]National Institute of Health Research, UCLH Biomedical Research Centre, London W1T 7DN, United Kingdom (UK).

Mr Kamal Mahawar, Consultant Surgeon, Sunderland Royal Hospital, Sunderland SR4 7TP

Dr Manisha Sharma, Consultant Chemical Pathologist, Bariatric & Lipid specialist, Homerton University Hospital NHS Trust, London

Prof Alex Blakemore, Department of Life Sciences, Brunel University, London, UK; Department of Medicine, Imperial College, London, UK

Iris McMillan, patient representative

Dr Julian H Barth, Consultant in Chemical Pathology & Metabolic Medicine, Leeds Teaching Hospitals NHS Trust

Corresponding author: Mary O'Kane, Dietetic Department, The General Infirmary at Leeds, Great George Street, Leeds, LS1 3EX

Email: Marywasokane@googlemail.com

Table S1 Search Strategy

Strategy 343036

# Database	Search term
1 Medline	exp "BARIATRIC SURGERY"/
2 Medline	(bariatric ADJ3 surg*).ti,ab
3 Medline	("gastric bypass").ti,ab
4 Medline	("gastric band*").ti,ab
5 Medline	("sleeve gastrectom*").ti,ab
6 Medline	("duodenal switch").ti,ab
7 Medline	(1 OR 2 OR 3 OR 4 OR 5 OR 6)
8 Medline	"POSTOPERATIVE CARE"/
9 Medline	(postoperative).ti,ab
10 Medline	(post-operative).ti,ab
11 Medline	(postsurg*).ti,ab
12 Medline	(post-surg*).ti,ab
13 Medline	(8 OR 9 OR 10 OR 11 OR 12)
14 Medline	exp "NUTRITION ASSESSMENT"/
15 Medline	exp "NUTRITION THERAPY"/
16 Medline	exp "NUTRITIONAL SUPPORT"/
17 Medline	exp "NUTRITIONAL PHYSIOLOGICAL PHENOMENA"/
18 Medline	exp "NUTRITIONAL REQUIREMENTS"/
19 Medline	exp AVITAMINOSIS/
20 Medline	("nutritional assessment*").ti,ab
21 Medline	("nutritional support").ti,ab
22 Medline	("nutritional requirement*").ti,ab
23 Medline	("nutritional correction*").ti,ab
24 Medline	("nutritional therap*").ti,ab
25 Medline	("nutritional treat*").ti,ab
26 Medline	("nutritional evaluation*").ti,ab
27 Medline	("vitamin deficienc*").ti,ab
28 Medline	(14 OR 15 OR 16 OR 17 OR 18 OR 19 OR 20

	OR 21 OR 22 OR 23 OR 24 OR 25
	OR 26 OR 27)
29 Medline	(7 AND 13 AND 28)
30 CINAHL	exp "GASTRIC BYPASS"/
31 CINAHL	exp "BARIATRIC SURGERY"/
32 CINAHL	(bariatric ADJ3 surg*).ti,ab
33 CINAHL	("gastric bypass").ti,ab
34 CINAHL	("gastric band*").ti,ab
35 CINAHL	("sleeve gastrectom*").ti,ab
36 CINAHL	("duodenal switch").ti,ab
37 CINAHL	(30 OR 31 OR 32 OR 33 OR 34 OR 35 OR 36)
38 CINAHL	exp "POSTOPERATIVE CARE"/
39 CINAHL	(postoperative).ti,ab
40 CINAHL	(post-operative).ti,ab
41 CINAHL	(postsurg*).ti,ab
42 CINAHL	(post-surg*).ti,ab
43 CINAHL	(38 OR 39 OR 40 OR 41 OR 42)
44 CINAHL	exp "NUTRITIONAL ASSESSMENT"/
45 CINAHL	exp "DIET THERAPY"/
46 CINAHL	exp "NUTRITIONAL SUPPORT"/
47 CINAHL	exp "NUTRITIONAL REQUIREMENTS"/
48 CINAHL	exp AVITAMINOSIS/
49 CINAHL	("nutritional assessment*").ti,ab
50 CINAHL	("nutritional support").ti,ab
51 CINAHL	("nutritional requirement*").ti,ab
52 CINAHL	("nutritional correction*").ti,ab
53 CINAHL	("nutritional therap*").ti,ab
54 CINAHL	("nutritional treat*").ti,ab
55 CINAHL	("nutritional evaluation*").ti,ab
56 CINAHL	("vitamin deficienc*").ti,ab
57 CINAHL	(44 OR 45 OR 46 OR 47 OR 48 OR 49 OR 50

	OR 51 OR 52 OR 53 OR 54 OR 55 OR 56)
58 CINAHL	(37 AND 43 AND 57)
59 EMBASE	exp "GASTRIC BANDING"/
60 EMBASE	exp "BARIATRIC SURGERY"/
61 EMBASE	(bariatric ADJ3 surg*).ti,ab
62 EMBASE	("gastric bypass").ti,ab
63 EMBASE	("gastric band*").ti,ab
64 EMBASE	("sleeve gastrectom*").ti,ab
65 EMBASE	("duodenal switch").ti,ab
66 EMBASE	(59 OR 60 OR 61 OR 62 OR 63 OR 64 OR 65)
67 EMBASE	exp "POSTOPERATIVE CARE"/
68 EMBASE	(postoperative).ti,ab
69 EMBASE	(post-operative).ti,ab
70 EMBASE	(postsurg*).ti,ab
71 EMBASE	(post-surg*).ti,ab
72 EMBASE	(67 OR 68 OR 69 OR 70 OR 71)
73 EMBASE	exp "NUTRITIONAL EVALUATION"/
74 EMBASE	exp "NUTRITIONAL DEFICIENCY"/
75 EMBASE	exp "NUTRITIONAL ASSESSMENT"/
76 EMBASE	exp "NUTRITIONAL SUPPORT"/
77 EMBASE	exp "NUTRITIONAL SUPPLEMENTATION"/
78 EMBASE	exp "NUTRITIONAL REQUIREMENT"/
79 EMBASE	exp "VITAMIN DEFICIENCY"/
80 EMBASE	("nutritional assessment*").ti,ab
81 EMBASE	("nutritional support").ti,ab
82 EMBASE	("nutritional requirement*").ti,ab
83 EMBASE	("nutritional correction*").ti,ab
84 EMBASE	("nutritional therap*").ti,ab
85 EMBASE	("nutritional treat*").ti,ab
86 EMBASE	("nutritional evaluation*").ti,ab

87 EMBASE	("vitamin deficienc*").ti,ab
88 EMBASE	(avitaminosis).ti,ab
89 EMBASE	(73 OR 74 OR 75 OR 76 OR 77 OR 78 OR 79 OR 80 OR 81 OR 82 OR 83 OR 84 OR 85 OR 86 OR 87 OR 88)
90 EMBASE	(66 AND 72 AND 89)
91 Medline	(7 AND 13 AND 28) [DT FROM 2014] [Languages English]
92 CINAHL	(37 AND 43 AND 57) [DT FROM 2014] [Languages eng]
93 EMBASE	(66 AND 72 AND 89) [DT FROM 2014] [English language]

Table S2 Quality Assessment Tools

Type of study	Risk of bias tool
systematic review	AMSTAR 2 (1)
narrative review	AMSTAR 2 (1)
randomised control trial	Cochrane Risk of Bias (2)
non-randomised control trial	Cochrane Risk of Bias (2)
survey study	Centre for Evidence-Based Medicine (CEBM)s Critical Analysis (3)
Cohort	Newcastle - Ottawa Quality Assessment Scale<br>Cohort Studies (4)
cross-sectional	Joanna Briggs Institute Critical Appraisal Checklists for Analytical Cross-sectional (5)
case control	Newcastle - Ottawa Quality Assessment Scale<br>Case Control Studies (4)
case study/report	Joanna Briggs Institute Critical Appraisal Checklists for Case Reports (6)
case series	Joanna Briggs Institute Critical Appraisal Checklist for Case Series (7)
case studies review	AMSTAR 2 (1)
clinical practice guidelines	Appraisal Of Guidelines For Research & Evaluation II (8)

Table S3: Full paper excluded studies with reasons for exclusions

Publication	Reason for exclusion
Ruiz-Tovar et al 2018 (9)	Focus is on food frequency. No relevant data
Freeman et al 2018 (10)	Patient group with condition that can lead to nutritional problems
Shoar et al 2017 (11)	No relevant nutritional data
Gomes et al 2017 (12)	No relevant nutritional data
Schiavo et al 2017 (13)	No relevant nutritional data
Marcotte et al 2016 (14)	Unable to obtain full paper
Taube-Schiff et al 2016 (15)	No relevant nutritional data
Schollenberger AE et al (16)	No relevant nutritional data
Beebe et al 2015 (17)	Unable to obtain full paper
Bose et al 2015 (18)	No relevant nutritional data
Leiro et al 2014 (19)	No relevant nutritional data
Lima TP et al 2014 (20)	Unable to obtain full paper
Gras-Miralles et al 2014 (21)	No relevant nutritional data
McGrice at al 2014 (22)	No relevant nutritional data
Walsh et al 2016 (23)	Not patients undergoing bariatric surgery
King et al 2017 (24)	Not specifically patients undergoing bariatric surgery
Klarenbach et al 2010 (25)	No relevant nutritional data
Fried et al 2014 (26)	Earlier version of included clinical guidance
Holick et al 2011 (27)	Not specifically patients undergoing bariatric surgery
Holick et al 2012 (28)	Not specifically patients undergoing bariatric surgery
Francis et al 2018 (29)	Not patients undergoing bariatric surgery
Elkins et al 2005 (30)	No relevant nutritional data
Sarwer et al 2008 (31)	No relevant nutritional data

Sarwer et al 2011 (32)	No relevant nutritional data
Welbourn et al 2018 (33)	No relevant nutritional data
Clees et al 2006 (34)	No relevant nutritional data
Baldry et al 2014 (35)	No relevant nutritional data
Nice Institute for Health and Care Excellence (NICE) CG32 2017 (36)	Not patients undergoing bariatric surgery
Sogg et al 2016 (37)	No relevant nutritional data
NICE Clinical Knowledge Summaries (CKS): Anaemia – iron deficiency 2018 (38)	Not patients undergoing bariatric surgery
NICE CKS: Anaemia - B12 and folate deficiency 2019 (39)	Not patients undergoing bariatric surgery
Jefferys et al 2013 (40)	No relevant nutritional data

References

1. Shea BJ, Reeves BC, Wells G, Thuku M, Hamel C, Moran J, Moher D, Tugwell P, Welch V, Kristjansson E, Henry DA. AMSTAR 2: a critical appraisal tool for systematic reviews that include randomised or non-randomised studies of healthcare interventions, or both. BMJ 2017;358:j4008
2. Higgins JPT, Savović J, Page MJ, Sterne JAC on behalf of the RoB2 Development Group Revised Cochrane risk-of-bias tool for randomized trials (RoB 2) 2019 Available from https://methods.cochrane.org/bias/resources/rob-2-revised-cochrane-risk-bias-tool-randomized-trials
3. Critical Appraisal of a Survey. Centre for Evidence-Based Medicine. Available from https://www.cebma.org/wp-content/uploads/Critical-Appraisal-Questions-for-a-Survey.pdf
4. Newcastle - Ottawa Quality Assessment Scale Available from http://www.ohri.ca/programs/clinical_epidemiology/nosgen.pdf
5. The Joanna Briggs Institute Critical Appraisal Checklist for Analytical Cross Sectional Studies 2017 Available from http://joannabriggs-webdev.org/assets/docs/critical-appraisal-tools/JBI_Critical_Appraisal-Checklist_for_Analytical_Cross_Sectional_Studies2017.pdf
6. The Joanna Briggs Institute Critical Appraisal Checklist for Case Reports 2017 Available from http://joannabriggs-webdev.org/assets/docs/critical-appraisal-tools/JBI_Critical_Appraisal-Checklist_for_Case_Reports2017.pdf
7. The Joanna Briggs Institute Critical Appraisal Checklist for Case Series 2017 Available from http://joannabriggs-webdev.org/assets/docs/critical-appraisal-tools/JBI_Critical_Appraisal-Checklist_for_Case_Series2017.pdf
8. Brouwers M, Kho ME, Browman GP, Cluzeau F, feder G, Fervers B, Hanna S, Makarski J on behalf of the AGREE Next Steps Consortium. AGREE II: Advancing guideline development, reporting and evaluation in healthcare. Can Med Assoc J. Dec 2010, 182:E839-842; doi: 10.1503/cmaj.090449
9. Ruiz-Tovar J, Bozhychko M, Del-Campo JM, Boix E, Zubiaga L, Munoz JL, Llavero C. Changes in Frequency Intake of Foods in Patients Undergoing Sleeve Gastrectomy and Following a Strict Dietary Control Obesity Surgery 2018; 28:1659–1664
10. Freeman LM, Strong AT, Sharma G, Punchai S, Rodriguez JH, Kroh M, Kirby DF. Implications of Celiac Disease Among Patients Undergoing Gastric Bypass Obesity Surgery 2018; 28:1546–1552
11. Shoar S, Mahmoudzadeh H, Naderan M, Bagheri-Hariri S, Wong C, Parizi AS, Shoar N. Long-Term Outcome of Bariatric Surgery in Morbidly Obese Adolescents: a Systematic Review and

Meta-Analysis of 950 Patients with a Minimum of 3 years Follow-Up. Obes Surg 2017; 27:3110–3117
12. Gomes DL, Moehlecke M, da Silva FBL, Dutra ES, D'Agord Schaan B, de Carvalho KMB.Whey Protein Supplementation Enhances Body Fat and Weight Loss in Women Long After Bariatric Surgery: a Randomized Controlled Trial. Obes Surg 2017; 27:424–431
13. Schiavo L, Scalera G, Pilone V, De Sena G, Ciorra FR, Barbarisi A. Patient adherence in following a prescribed diet and micronutrient supplements after laparoscopic sleeve gastrectomy: our experience during 1 year of follow-up. J Hum Nutr Diet. 2017: 30, 98–104
14. Marcotte Eric, Bipan C. Management and Prevention of Surgical and Nutritional Complications After Bariatric Surgery. Surgical Clinics 2016; 96:843–856
15. Taube-Schiff M, Chaparro M, Gougeon L, Shakory S, Weiland M, Warwick K, Plummer C, Sockalingam S. Examining Nutrition Knowledge of Bariatric Surgery Patients: What Happens to Dietary Knowledge over Time? Obes Surg 2016; 26:972–982
16. Schollenberger AE, Karschin J, Meile T, Küper MA, Königsrainer A, Bischoff SC. Impact of protein supplementation after bariatric surgery: A randomized controlled double-blind pilot study. - Nutrition. 2016; 32:186-92. doi: 10.1016/j.nut.2015.08.005
17. Beebe ML, Crowley N. Can Hypocaloric, High-Protein Nutrition Support Be Used in Complicated Bariatric Patients to Promote Weight Loss? Nutrition in Clinical Practice 2015; 30: 522-529
18. Bose S, Khanna A, You J, Arora L, Qavi S, Turan A. Low serum vitamin D levels are not associated with increased postoperative pain and opioid requirements: a historical cohort study. Canadian journal of anaesthesia = Journal canadien d'anesthesie. 2015; 62:770-776
19. Leiro LS, Melendez-Araújo MS Diet micronutrient adequacy of women after 1 year of gastric bypass. Arq Bras Cir Dig 2014;27 (1):21-25 Effect of weight loss on bone mineral density determined by ultrasound of phalanges in obese women after Roux-en-y gastric bypass: conflicting results with dual-energy X-ray absorptiometry.
20. Lima TP, Nicoletti CF, Marchini JS, Junior WS, Nonino CB. Effect of weight loss on bone mineral density determined by ultrasound of phalanges in obese women after Roux-en-y gastric bypass: conflicting results with dual-energy X-ray absorptiometry. Journal of clinical densitometry 2014; 17:473-478
21. Gras-Miralles B, Haya JR, Moros JMR, Arnó A G, Alsina S T, Sánchez LI, Galitó JM, Zafón II, Romera MCA, Bonet AP, Ros FB, Garcia MA, Delgado-Aros S. Caloric intake capacity as measured by a standard nutrient drink test helps to predict weight loss after bariatric surgery. Obes Surg 2014; 24:2138–2144
22. McGrice MA, Porter JA. The micronutrient intake profile of a multicentre cohort of Australian LAGB patients. Obes Surg 2014; 24:400–404
23. Walsh JS, Evans AL, Bowles S, Naylor KE, Jones KS, Schoenmakers I, Jacques RM, Eastell R. Free 25-hydroxyvitamin D is low in obesity, but there are no adverse associations with bone health Am J Clin Nutr. 2016 Jun;103(6):1465-71
24. King RJ, Chandrajay D, Abbas A, Orme SM, Barth JH. High-dose oral colecalciferol loading in obesity: impact of body mass index and its utility prior to bariatric surgery to treat vitamin D deficiency. Clin Obes. 2017; 7:92-97. not bariatric surgery patients
25. Klarenbach S, Padwal R, Wiebe N, Hazel M, Birch D, Manns B et al. (2010) Bariatric surgery for severe obesity: systematic review and economic evaluation, technology report; no. 129 [Internet], Ottawa, Canadian Agency for Drugs and Technologies in Health. 2010. Available from: http://www.cadth.ca/index.php/en/hta/reports- publications/search?&type=16
26. Fried M, Yumuk V, Oppert JM, Scopinaro N, Torres A, Weiner R et al. on behalf of International Federation for the Surgery of Obesity and Metabolic Disorders—European

Chapter (IFSO-EC) and European Association for the Study of Obesity (EASO). Interdisciplinary European guidelines on metabolic and bariatric surgery. Obes Surg. 2014; 24:42–55

27. Holick MF, Binkley NC, Bischoff-Ferrari HA, Gordon CM, Hanley DA, Heaney RP et al. Evaluation, treatment, and prevention of vitamin D deficiency: an Endocrine Society clinical practice guideline. J Clin Endocrinol Metab. 2011; 96: 1911-30
28. Holick MF, Binkley NC, Bischoff-Ferrari HA, Gordon CM, Hanley DA, Heaney RP et al. Guidelines for preventing and treating vitamin D deficiency and insufficiency revisited. J Clin Endocrinol Metab. 2012; 97(4):1153–1158
29. Francis R, Aspray T, Fraser W, Macdonald H, Patel S, Mavroeidi A, Schoenmakers I, Stone M. National Osteoporosis Society Vitamin D and Bone Health: A Practical Clinical Guideline for Patient Management 2018 Available from https://theros.org.uk/media/100231/nos_vitamin_d_and_bone_-health_in_adults_web.pdf
30. Elkins G, Whitfield P, Marcus J, Symmonds R, Rodriguez J, Cook T. Noncompliance with behavioural recommendations Following Bariatric Surgery. Obes Surg. 2005; 15(4): 546-551.
31. Sarwer DB, Wadden TA, Moore RH, Baker AW, Gibbons LM, Raper SE et al. Preoperative eating behaviour, postoperative dietary adherence, and weight loss after gastric bypass surgery. Surg Obes Relat Dis 2008; 4(5):640-646.
32. Sarwer DB, Dilks RJ, West-Smith L. Dietary intake and eating behaviour after bariatric surgery: threats to weight loss maintenance and strategies for success. Surg Obes Relat Dis 2011; 7(5):644-651.
33. Welbourn R, Hopkins J, Dixon JB, Finer N, Hughes C, Viner R, Wass J8 and on behalf of the Guidance Development Group. Commissioning guidance for weight assessment and management in adults and children with severe complex obesity. Obesity Reviews 19, 14–27, January 2018
34. Colles SL, Dixon JB, Marks P, Strauss BJ, O'Brien PE. Preoperative weight loss with a very-low-energy diet: quantitation of changes in liver and abdominal fat by serial imaging. Am J Clin Nutr. 2006; 84: 304-11
35. Baldry EL, Leeder PC, Idris IR. Pre-operative dietary restriction for patients undergoing bariatric surgery in the UK: observational study of current practice and dietary effects. Obes Surg. 2014; 24:416-421
36. National Institute for Health and Clinical Excellence. NICE CG32 Nutrition support for adults: oral nutrition support, enteral tube feeding and parenteral nutrition [Internet] 2017. Available from: https://www.nice.org.uk/guidance/cg32/chapter/1-Guidance . [Accessed 1 September 2018]
37. Sogg S, Lauretti J, West-Smith L. ASMBS Guidelines/Statements Recommendations for the presurgical psychosocial evaluation of bariatric surgery patients. Surg Obes Relat Dis 2016; 12: 731–749
38. NICE CKK: Anaemia – iron deficiency 2018 Available from https://cks.nice.org.uk/anaemia-iron-deficiency patients
39. NICE CKS: Anaemia - B12 and folate deficiency 2019 Available from https://cks.nice.org.uk/anaemia-b12-and-folate-deficiency
40. Jefferys AE, Siassakos D, Draycott T, Akande VA, Fox R Deflation of gastric band balloon in pregnancy for improving outcomes. no relevant nutritional data. Cochrane Database of Systematic Reviews 2013, Issue 4. Art. No.: CD010048. DOI: 10.1002/14651858.CD010048.pub2.
